# Investigation of the La–Al–H and La–Si–H Systems at High Pressures

**DOI:** 10.1021/acs.inorgchem.5c05140

**Published:** 2026-01-22

**Authors:** Doreen C. Beyer, Pedro Nunes Ferreira, Roman Lucrezi, Luiz Tadeu Fernandes Eleno, Holger Kohlmann, Christoph Heil, Michael Sannemo Targama, Volodymyr Baran, Shrikant Bhat, Robert Farla, Kristina Spektor, Ulrich Häussermann

**Affiliations:** † Institute of Inorganic Chemistry and Crystallography, Faculty of Chemistry, 9180 Leipzig University, Johannisallee 29, D-04103 Leipzig, Germany; ‡ DEMAR, Escola de Engenharia de Lorena, 119119 Universidade de São Paulo, 12612-550 Lorena, Brazil; § Institute of Theoretical and Computational Physics, 27253 Graz University of Technology, NAWI Graz, 8010 Graz, Austria; ∥ Department of Chemistry, 7675 Stockholm University, 10691 Stockholm, Sweden; ⊥ 28332 Deutsches Elektronen-Synchrotron DESY, Notkestraße 85, D-22607 Hamburg, Germany

## Abstract

Hydrogenation at gigapascal pressures can produce hydrides with potential superconducting, ionic, and hydrogen-storage properties. We studied the La–Al–H and La–Si–H systems up to 20 GPa using structure prediction and *in situ* synchrotron diffraction. In La–Al–H, only rhombohedral LaAlH_6_ is stable. The La–Si–H system forms an orthorhombic monohydride, LaSiH, at low pressure, while LaSiH_2_ and LaSiH_7_ are predicted to be stable at 20 GPa, and LaSiH_6_ is slightly unstable. LaSiH_2_ is structurally related to the monohydride, whereas LaSiH_6_ and LaSiH_7_ feature SiH_6_^2–^ units characteristic of hydridosilicates. Calculations predict superconductivity in LaSiH_2_ and LaSiH_6_ with *T*_c_ ≈ 10 and 6 K. Experimentally, LaSiH_2_ formation is indicated at 20 GPa, but higher hydrides were not observed due to decomposition into LaH_3_ and Si, suggesting that pressures above 20 GPa are required to stabilize these phases at synthesis temperatures.

## Graphical abstract

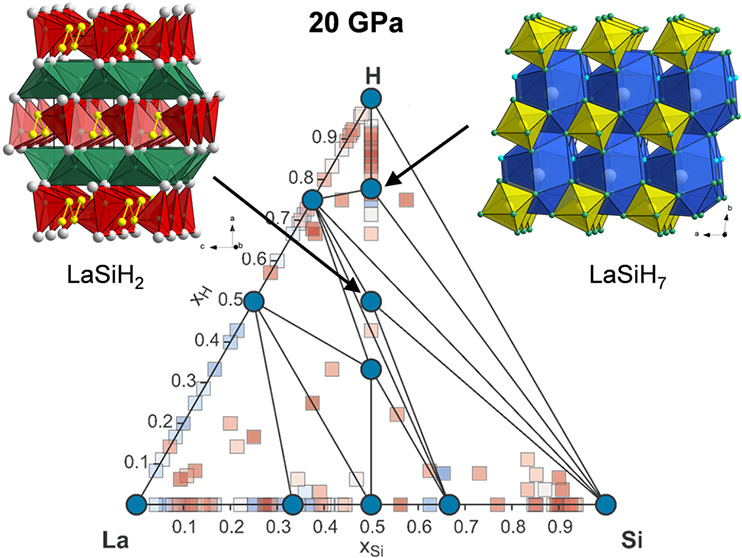

## 1 Introduction

The lanthanum–hydrogen system has attracted significant attention following the report of near room temperature superconductivity in LaH_10_ at pressures above 150 GPa.^1,2^ Subsequent studies elaborated on this finding^3^ and stimulated the investigation of numerous other *M*–H systems (e.g., M = Ca, Ce) from which a larger class of potentially high temperature superconducting (H*T*_c_) hydrides emerged.^4–7^ Yet these so-called superhydrides are only observable *in situ* at highly extreme conditions and as minute samples, which makes their characterization extremely challenging.^8^ Comprehensive and conclusive studies of the H*T*_c_ phenomenon and, importantly, its potential exploitation as useful materials property would require that superhydrides can be retained as larger sample quantities at lower pressures, ideally at ambient pressure.^9,10^

With LaH_10_ as the starting point, design principles for robust ternary derivatives with H*T*_c_ properties have been suggested,^11,12^ with the term “robust” referring to dynamic stability at pressures considerably lower than required for thermodynamic stability.^13,14^ Dynamic stability indicates that a structure is in a local minimum of the potential energy surface. To actually retain a superhydride at low pressures would also require its kinetic stability toward decomposition into more stable configurations, i.e., sufficiently high potential energy barriers. Compared to binaries, ternary/multinary materials are expected to possess higher kinetic stability because of more complex decomposition pathways.^15^ From theoretical works, LaBH_8_ has been identified as dynamically stable down to 40 GPa and with a *T*_c_ of 126 K at 50 GPa.^13^ For LaBeH_8_, dynamic stability was calculated even down to 20 GPa, but experiments showed kinetic stability only to 80 GPa (at which LaBeH_8_ displays a *T*_c_ of 110 K).^16^ LaB_2_H_8_ has been shown kinetically stable down to 60 GPa and with a *T*_c_ of 106 K at 90 GPa.^17^ Ultimately, for obtaining useful sample quantities for conclusive property characterization, it will be important to identify materials that are also thermodynamically stable at pressures down to 20–30 GPa.^9^

Against this background, we pursued an investigation of the La–Al–Si and La–Si–H systems by crystal structure prediction (CSP) and performed *in situ* studies of hydrogenations of the intermetallic samples LaAl, LaSi, and LaAl_0.5_Si_0.5_ at pressures up to 20 GPa. For this, we employed large volume press (LVP) high pressure methodology.^18^ In contrast with diamond anvil cell (DAC) devices, pressures in LVP hydrogenations are limited to 20–25 GPa (and thus LVP techniques would not allow for the synthesis of LaH_10_). In exchange, sample volumes are drastically increased (to tens of mm^3^) and reaction environments at high *p*, *T* can be stably maintained and well controlled over prolonged periods of time. We find that LaAl can be hydrogenated to LaAlH_6_ at very low pressures, 2 GPa, and that LaAl_0.5_Si_0.5_ decomposes to LaAlH_6_ + Si. CSP suggests thermodynamic stability for the previously unknown hydrides LaSiH_2_ and LaSiH_7_ at 20 GPa. The former material corresponds to a metal with a predicted *T*_c_ of around 10 K, whereas LaSiH_7_ represents a semiconducting hydridosilicate with SiH_6_^2–^ complexes. However, hydrogenation of LaSi at 20 GPa and temperatures around 500 °C resulted in decomposition to LaH_3_ + Si, suggesting that higher pressures (outside the reach of LVP techniques) are needed for stabilizing these hydrides at synthesis temperatures.

## 2 Experimental and Computational Details

### 2.1 Precursor Synthesis and Characterization

All steps of synthesis and preparations for sample characterization were carried out in a glovebox under an Ar atmosphere. Precursors with (nominal) compositions LaAl, LaSi, and LaAl_0.5_Si_0.5_ were prepared by arc melting 1–5 g batches of stoichiometric amounts of constituting elements (La, Chempur with a purity of 99.9% or better; Si, ABCR GmbH, 99,9999%; Al shots, ABCR GmbH, 99.999%). The samples were flipped over and remelted several times to ensure homogeneity. The total mass loss was negligible (<0.4 wt %). The obtained materials did not show noticeable degradation when exposed to air. The LaSi precursor corresponded to almost phase pure intermetallic compound LaSi with the orthorhombic FeB structure (*Pnma*),^19^ whereas LaAl and LaAl_0.5_Si_0.5_ represented multiphase mixtures (LaAl, LaAl_2_, La_5_Al_4_, La_2_Al and LaAl_0.45_Si_0.55_, La­(Al_0.88_Si_0.12_)_2_, La_5_(Al_0.81_Si_0.19_)_4_, respectively) with an overall composition close to the nominal synthesis composition. For simplicity, we refer to these precursors in the following as LaAl and LaAl_0.5_Si_0.5_. See Supporting Information for details on the analysis of the precursor materials.

### 2.2 High Pressure Experiments and Data Analysis

All steps of sample preparation and recovery were performed in a glovebox under an Ar atmosphere. Powdered LaAl, LaSi, and LaAl_0.5_Si_0.5_ were compressed into pellets with a diameter of 1.0 mm and a height of 0.6–0.8 mm. In LVP hydrogenation, H_2_ has to be delivered by a chemical source, which is integrated in the sample and releases H-fluid at the targeted *p*, *T* conditions.^18^ Ammonia borane, BH_3_NH_3_, has emerged as preferred H-source as it possesses a high H content and decomposes neatly to inert BN and H_2_ at high pressures.^20^ The amount of BH_3_NH_3_ (ABCR, 97%) used for each sample corresponded to a molar ratio H_2_:La of at least 3:1 (cf. Table 1). Precursor sample pellets were sandwiched between pelletized BH_3_NH_3_ and sealed inside NaCl capsules with 3.0 mm (14/7) or 2.5 mm (10/4) OD, according to established procedures.^21–23^

**Table 1:** Compilation of Performed Experiments and Applied Conditions

run	precursor	target pressure, GPa	*T*_max_, °C	total heating duration, h	H content, assembly
#1, BT829	LaAl	10.2 → 12.1	490–495 → 630	∼4.8	×8H, 14/7
#2, BT832	LaAl	2.2	490	∼4.5	×8H, 14/7
#3, BT652	LaSi	9.0	570	∼5.14	×6H, 14/7
#4, BT830	LaSi	20.0	800	∼4.9	×8H, 10/4
#5, BT831	LaAl_0.5_Si_0.5_	9.3	615	∼6.9	×8H, 14/7

*In situ* high pressure experiments were performed at approximately 2 and 10–12 GPa for LaAl, 9 and 20 GPa for LaSi, and 9 GPa for LaAl_0.5_Si_0.5_ (cf. Table 1) and utilized the standard multianvil assemblies employed at the LVP beamline P61B, PETRA III, DESY (14/7 and 10/4 for pressures below and above 15 GPa, respectively).^24^ For 10/4 assemblies, a 4.8 mm high TiB_2_ heater (3.2 mm OD/2.5 mm ID), ZrO_2_ with 3.2 mm OD, and NaCl capsules and MgO plugs with 2.5 mm OD were employed. Assemblies were compressed to target pressure and initially heated to a temperature between 250 and 350 °C at which the H-source BH_3_NH_3_ is expected to decompose and release hydrogen fluid.^20^ The samples were then equilibrated for about 15–20 min before further heating. The temperature was evaluated from the power-*T* calibration curves. Energy-dispersive XRD (EDXRD) patterns were collected using two germanium solid-state detectors positioned at around 3 and 5°, respectively. Angle calibration was performed using LaB_6_ SRM 660c. Initial data evaluation and manipulation utilized PDIndexer.^25^ The Le Bail analysis^26^ of the *in situ* EDXRD data was performed in GSAS-II.^27^ Pressure was estimated from reflections of the sample capsule and using the equation of state of NaCl by Matsui et al.^28^

### 2.3 *Ex Situ* PXRD Characterization of La–Al/Si–H Products

The products obtained from runs #1, #3, and #5 were recovered in a glovebox under an argon atmosphere. Approximately half of each sample pellet was sealed inside a 1.0 mm wide glass capillary. PXRD patterns were collected at the beamline P02.1, PETRA III, DESY^29^ using monochromatic synchrotron radiation (λ = 0.20730 Å, *E* ≈ 60 keV) at ambient *p*, *T*. Data was collected on spinning samples (∼5000 Hz) using the Varex XRD 4343CT (150 × 150 μm^2^ pixel size, 2880 × 2880 pixel area) detector and integrated using the pyFAI software.^30^ Calibration was performed based on data collected for LaB_6_ powder (SRM 660c). For indexing of the angle-dispersive powder patterns, the DICVOL^31^ and TAUP algorithms^32^ within the CRYSFIRE package were used.^33^ Rietveld refinements^34^ of the *ex situ* PXRD data were performed in Fullprof.^35^ Formation of lanthanum hydride was observed during the experiments. Since in all the experiments a H_2_:La ratio of at least 3:1 was employed, a stoichiometric formation of LaH_3_ was assumed.^36^ With the techniques used, LaH_3_ could not clearly be distinguished from a mixed stoichiometry LaH_2+*x*_ (0 ≤ *x* ≤ 1). For Rietveld refinements of the recovered samples, a *Fm*3̅*m* LaH_3_ structure was used.

### 2.4 Theoretical Calculations

The USPEX package^37,38^ was employed for evolutionary CSP to sample the phase space of possible candidate structures in the ternary systems of La–Al–H and La–Si–H. For each pressure, 0 and 20 GPa, several independent USPEX runs were performed with variable composition (VC) according to (i) the binary boundary lines La–Al, La–Si, Si–H, Al–H, and La–H, (ii) the full ternary space, and (iii) the pseudobinary lines LaSi–H and LaAl–H. All VC runs were restricted to unit cells containing 16–32 atoms. Additional fixed-composition (FC) runs were performed at 0 and 20 GPa for the stable or nearly stable compositions determined in the VC runs: LaSiH, LaSiH_6_, and LaSiH_7_. For each FC, we carried out three separate runs with the number of atoms per cell restricted to 6–12, 12–24, 24–48 for LaSiH; 8–16, 16–32, and 24–48 for LaSiH_6_; 9–18, 18–36, 27–54 for LaSiH_7_. For the composition of LaSiH_2_, no stable structure was found within the VC runs and two candidates, determined by chemical intuition, were added by hand. Within the described USPEX runs, we sampled a total of approximately 10,000 (15,000) structures per pressure for the ternary La–Al–H (La–Si–H) systems.

The geometry optimization (relaxation) of suggested candidate structures, as well as the calculation of energies, forces, and stress tensors, were carried out within density-functional theory (DFT) as implemented in the VASP code,^39,40^ using projector-augmented-wave (PAW)^41^ pseudopotentials and the Perdew–Burke–Ernzerhof (PBE)^42^ exchange-correlation functional. For each structural relaxation, we employed a five-step procedure with gradually increasing numerical parameters and convergence criteria, up to a final step with an energy cutoff for the plane-wave basis set of 375 eV, a **k**-grid spacing of 0.04 2π Å^–1^, a smearing value of 0.06 eV, and a self-consistency threshold of 10^–6^ eV.

For selected structures, we performed additional DFT calculations via the Quantum ESPRESSO (QE) package.^43–45^ Vibrational properties and electron–phonon coupling quantities were calculated within density-functional perturbation theory (DFPT).^46^ We used scalar-relativistic optimized norm-conserving Vanderbilt (ONCV) pseudopotentials from the SG15 ONCV library^47,48^ in combination with the PBE^42^ exchange-correlation functional. The kinetic energy cutoff for the wave functions was set to 100 Ry, the **k**-grid spacing to 0.02 2π Å^–1^, the convergence threshold for the electronic self-consistency to 10^–10^ Ry, and a Methfessel-Paxton smearing^49^ value of 0.01 Ry was used for metallic structures. These settings provide a numerical accuracy for the total energy of well below 1 meV/atom with respect to selected reference calculations with an energy cutoff of 300 Ry and a **k**-grid spacing of 1/3 0.02 2π Å^–1^. The Henkelman-group approach and code^50–52^ was used for the Bader analysis.^53^ The required charge-densities were calculated using kjPAW^54^ pseudopotentials from the PSLibrary^55^ employing a kinetic energy cutoff of 140 Ry for the wave functions and 1120 Ry for the charge-density.

The phonon self-consistency threshold was set to 10^–16^, and the employed **q**-grids were 4 × 4 × 4 for LaSiH and LaSiH_6_, 2 × 4 × 4 for LaSiH_2_, and 2 × 2 × 2 for LaSiH_7_. The electron–phonon matrix elements were integrated over a denser **k**-grid with spacing of 0.01 2πÅ^–1^ and for 20 double-delta smearing values in the range of 0.002–0.040 Ry, where a value of 0.010 Ry was chosen for the presented results in combination with a phonon smearing value of 0.2 THz for the **q**-grid integration. Based on the obtained Eliashberg spectral function α^2^ *F*(ω), the electron–phonon coupling strength λ and the logarithmic average phonon frequency ω_log_ are calculated according to

$$\lambda \left(\omega \right)=2\int_{0}^{\omega }d\omega^{\prime }\;\frac{\alpha^{2}F\left(\omega^{\prime }\right)}{\omega^{\prime }}$$

with λ = λ(∞) and

$$\omega_{\log}=\exp\left(\frac{2}{\lambda }\int_{0}^{\infty }d\omega \;\frac{\alpha^{2}F\left(\omega \right)\;\ln\omega }{\omega }\right)$$

The *T*_c_ values are obtained by solving the isotropic Migdal-Eliashberg (ME) equations in the full-bandwidth formulation using the IsoME package^56^ and a typical value for the Morel-Anderson pseudopotential^57^ of μ* = 0.1 (specified as μ^AD^ in IsoME).

## 3 Results and Discussion

### 3.1 Crystal Structure Prediction (CSP)

The results from CSP for the ternary systems La–Al–H and La–Si–H for 0 and 20 GPa are compiled in Figures 1 and 2, respectively. For La–Al–H, only previously known LaAlH_6_ is a stable ternary compound. The LaAlH_6_ structure contains isolated [AlH_6_]^3–^ octahedra and was reported with a *R*3̅*m* structure (BaSiF_6_ type),^58^ Figure 3a. La and Al atoms form a rhombohedrally distorted CsCl structure, which implies that AlH_6_^3–^ octahedra are surrounded by a rhombically distorted cube of La atoms. La atoms are 12-coordinated by H atoms in a cuboctahedral fashion. We find that a lower symmetric *R*3̅ structure is more stable, very slightly at ambient pressure and increasingly with pressure. This is depicted in Figure S1. The symmetry lowering is shown in Figure 3b. It is considered unlikely that additional ternary La–Al–H hydrides can be synthesized at pressures accessible with LVPs; instead substantially higher pressures would be required. A superconducting solid solution (La,Al)­H_10_ being stable at 146 GPa has been recently reported.^59^

**Figure 1:**
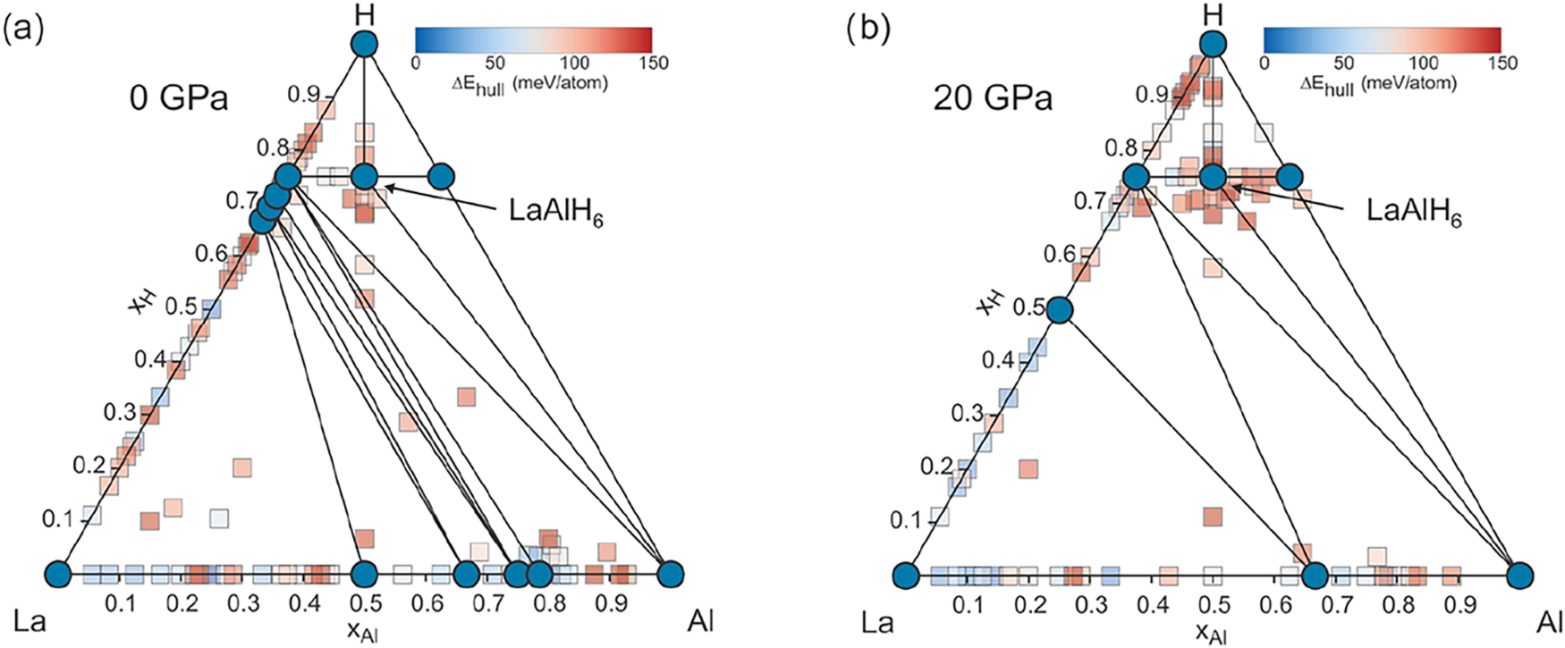
La–Al–H ternary phase diagram according to CSP at 0 (a) and 20 GPa (b). Blue circles represent compounds located on the convex hulls. Square symbols denote phases above the convex hulls. The colors of the squares (ranging from blue to red) indicate the magnitude of instability.

**Figure 2:**
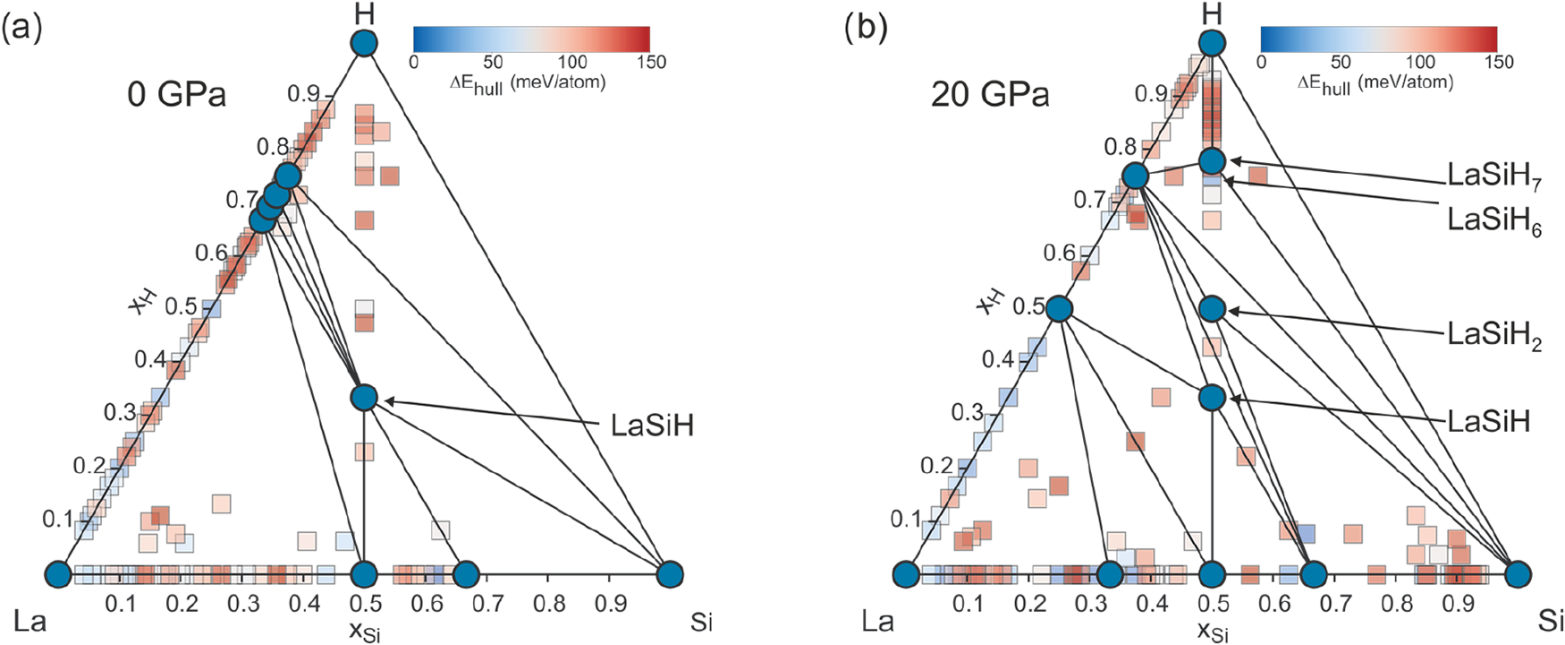
La–Si–H ternary phase diagram according to CSP at 0 (a) and 20 GPa (b). Blue circles represent compounds located on the convex hulls. Square symbols denote phases above the convex hulls. The color of the squares (ranging from blue to red) indicates the magnitude of instability.

**Figure 3:**
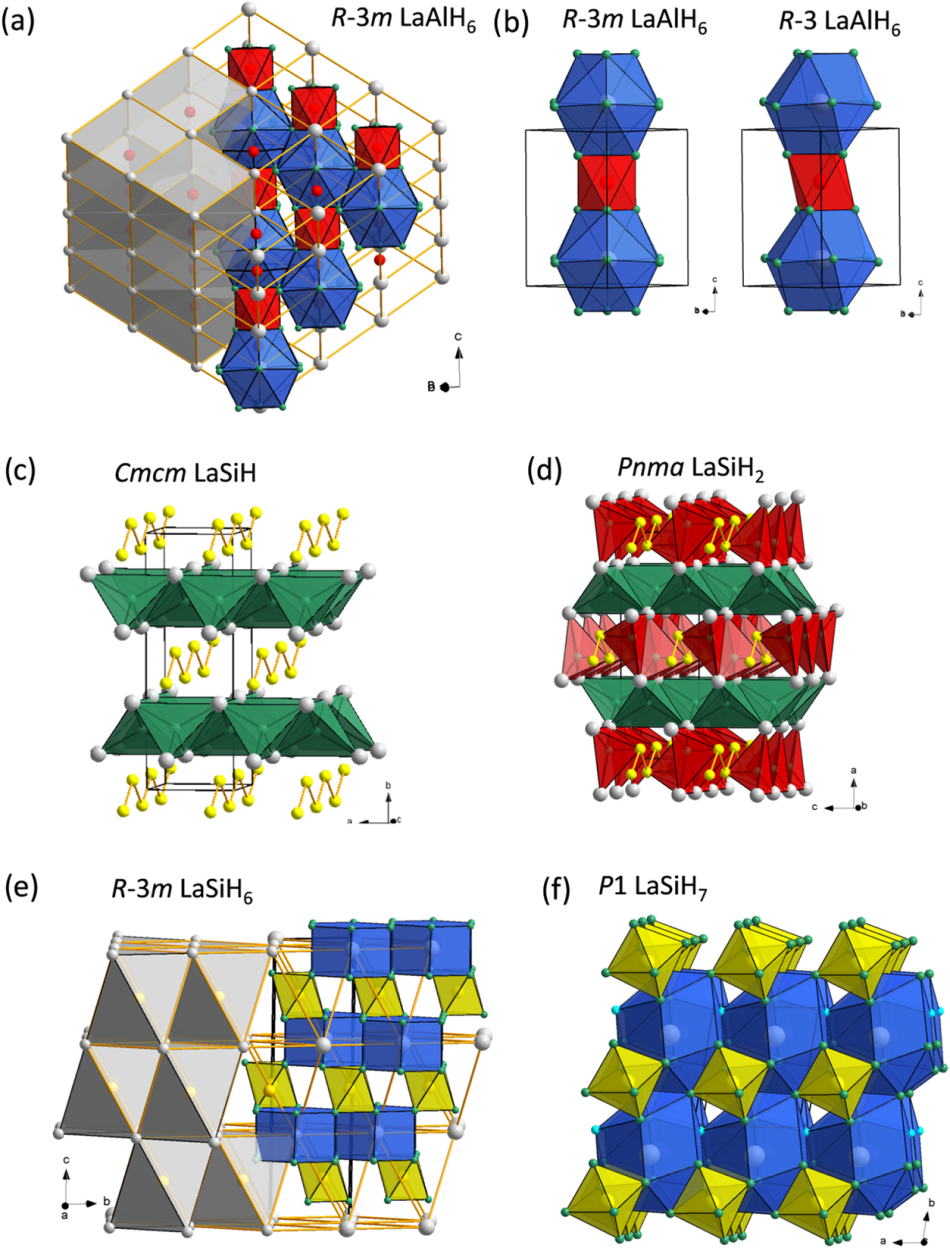
(a) Crystal structure of *R*3̅*m* LaAlH_6_, highlighting the CsCl-type arrangement of La^3+^ and AlH_6_^3–^ ions. The cuboctahedral and octahedral coordination for La and Al by H atoms, respectively, are shown as blue and red polyhedra. (b) Comparison of the arrangement of LaH_12_ and AlH_6_ polyhedra in *R*3̅*m* and slightly more stable *R*3̅ LaAlH_6_. (c) Crystal structure of the interstitial hydride *Cmcm* LaSiH, which closely relates to the CrB structure type. Tetrahedral interstitials defined by 4 La atoms and filled by H atoms are depicted as green polyhedra. (d) Crystal structure of *Pnma* LaSiH_2_. Compared to LaSiH, a second type of tetrahedral interstices defined by 3 La and 1 Si atoms is filled by H atoms (red polyhedra). (e) Rhombohedral crystal structure of metastable LaSiH_6_, highlighting the NaCl arrangement of La^3+^ and SiH_6_^2–^ ions. The hexagonal prismatic and octahedral coordinations for La and Si by H atoms, respectively, are shown as blue and yellow polyhedra. (f) Crystal structure of *P*1 LaSiH_7_. La is coordinated irregularly by 14 H atoms (depicted as blue polyhedra).

For La–Si–H at ambient pressure (Figure 2a), the interstitial hydride LaSiH with a *Cmcm* structure (Figure 3c) represents the only stable ternary compound. Also, this compound, although H-deficient, has been reported earlier.^60,61^ LaSiH_*x*_ with *x* = 0.6–0.9 was obtained as a dimorphic mixture of related *Cmcm* and *Pnma* phases in close to ambient pressure hydrogenations of LaSi.^61^ According to DFT calculations, the *Cmcm* form is slightly more favorable, by around 18 meV/atom at ambient pressure, and remains so, although increasingly less with pressure, cf. Figure S2. LaSiH features polyanionic zigzag chains of Si atoms (_∞_^1^[Si^2–^]) and hydridic H (H^–^) incorporated in tetrahedral La_4_ interstices and may be formally considered as charge-balanced Zintl phase hydride La^3+^(Si^2–^)­(H^–^).^61^

The La–Si–H diagram at 20 GPa looks noticeably different by revealing additionally the stable hydrides LaSiH_2_ and LaSiH_7_ (Figure 2b). LaSiH_2_ has a *Pnma* structure which resembles that of BaSiH_2_.^62^ This structure can be derived from *Cmcm* LaSiH by additional H occupying a second kind of tetrahedral interstice defined by three La atoms and one Si atom (La_3_Si), as shown in Figure 3d. In contrast with LaSiH, LaSiH_2_ would not correspond to a charge-balanced compound. LaSiH_7_ is a hydridosilicate. Its structure is depicted in Figure 3f. The characteristic of hydridosilicates is hypervalent octahedral SiH_6_^2–^ moieties. LaSiH_7_ appears charge-balanced due to the simultaneous presence of the H^–^ ion. Interestingly, the composition LaSiH_6_ also seems feasible. A hydridosilicate with *R*3̅*m* structure (Figure 3e) is just slightly above the ternary hull (by 18 meV/atom), and thus could potentially be stable at finite temperatures or be accessible as a metastable phase.^63^ Like LaSiH_2_, LaSiH_6_ would be charge-imbalanced and represent a metal.

Hydridosilicates have been recently established with alkali and alkaline earth metal counterions, e.g., K_2_SiH_6_, BaSiH_6_, from high pressure hydrogenations at 4–10 GPa.^23,64–66^ Hitherto, these materials have been exclusively found as charge-balanced, semiconducting compounds. Here, the CSP indicates a possible extension of hydridosilicates toward trivalent lanthanide counterions. In the *P*1 LaSiH_7_ structure, La ions are irregularly coordinated by 14 H atoms. La ions and SiH_6_^2–^ moieties attain a mutually tetrahedral coordination (relating to the wurtzite structure). In the *R*3̅*m* LaSiH_6_ structure, La ions are hexagonal-prismatically coordinated by 12 H atoms. La ions and SiH_6_^2–^ moieties are arranged as in the cubic NaCl structure, which is similar to the arrangement of constituents in BaSiH_6_ ^66^ but contrasts with the CsCl-like arrangement of La ions and AlH_6_^3–^ moieties in the LaAlH_6_ structure, see Figure 3a. The electronic structure and dynamic stability of the ternary LaSiH_n_ compounds will be discussed in more detail in Section 3.3.

### 3.2 *In Situ* Experiments

#### 3.2.1 LaAl

High pressure hydrogenations of LaAl were performed at 2 and 10–12 GPa. Figure 4 shows the evolution of EDXRD patterns during heating to 490 °C at 10 GPa, further compression to 12 GPa, and final heating to 630 °C. After hydrogen release at ∼300 °C and up to ∼355 °C, broad diffraction peaks in the energy range of 75–100 keV indicated the formation of intermediate hydride phases. At ∼390 °,C reflections of rhombohedral LaAlH_6_ appeared. The phase was stable upon further heating to 490 °C at 10 GPa and up to 630 °C after further pressure increase to 12 GPa. After cooling and decompression, *R*3̅*m* LaAlH_6_ was recovered to ambient conditions. Its PXRD pattern, shown in Figure 5, revealed the presence of a minute amount of LaH_3_ (about 5% with respect to LaAlH_6_), indicating the onset of decomposition above 600 °C and 12 GPa. Theory suggested that a less symmetric *R*3̅ structure is more stable. Since the symmetry lowering essentially relates to the H atom arrangement, possibly only neutron diffraction (on deuterated samples) may reveal the actual structure of LaAlH_6_.

**Figure 4:**
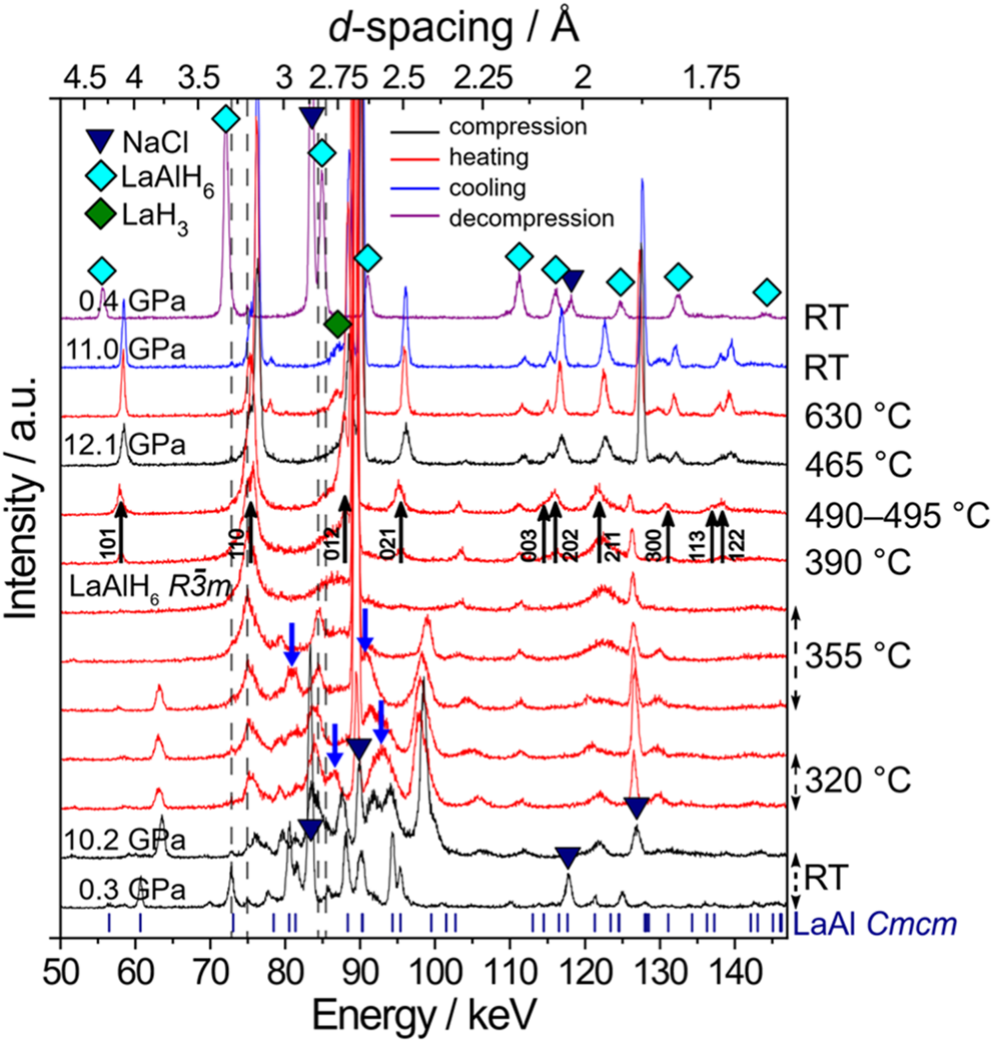
Hydrogenation of LaAl at 10–12 GPa. Diffraction patterns are shown for the starting and initial target pressure (10.2 GPa, black), during heating to 490 °C (red), further compression to 12.1 GPa (black), and further heating to 630 °C (red), after cooling to room temperature (blue), and after decompression (purple). Secondary Pb fluorescence peaks (from the detector shielding) are marked as dashed gray vertical lines, and blue triangles mark NaCl reflections from the sample capsule. The evolution of intermediate ternary hydride phases between hydrogen release at ∼320 °C and heating to 355 °C is indicated by blue arrows. The onset of LaAlH_6_ formation at 390 °C is marked by black arrows.

LaAlH_6_ was previously obtained from a mechanochemically assisted metathesis reaction between LaCl_3_ and NaAlH_4_, according to LaCl_3_ + 3NaAlH_4_ → LaAlH_6_ + 3NaCl + 2Al + 3H_2_, using a 3× excess of NaAlH_4_ and employing a slightly pressurized hydrogen atmosphere (1–15 bar).^58^ The reaction produced side products (Al and NaCl) and required several hours for completion. Here, we obtained LaAlH_6_ from direct synthesis by hydrogenating the intermetallic compound mixture “LaAl”. Hydrogenation attempted at the lowest possible pressures, around 2 GPa, showed LaAlH_6_ formation at 270 °C, see Figure S3. When heating to 490 °C, decomposition to LaH_3_ + Al was observed. It may be possible that LaAlH_6_ can also be synthesized by conventional autoclave hydrogenations using pressures 10–100 bar. The lattice parameters of LaAlH_6_ at various *p*, *T* conditions are compiled in Table S1.

#### 3.2.2 LaAl_0.5_Si_0.5_

High pressure hydrogenation of the LaAl_0.5_Si_0.5_ precursor was performed at about 9.3 GPa, Figure S4. An intermediate hydride LaAl_0.5_Si_0.5_H_*x*_ (with *Cmcm* structure and relating to LaSiH, cf. Figure 3c) may be inferred upon dwelling the sample at 320 °C, see Figure S5. The formation of LaAlH_6_, obviously accompanied by the decomposition of LaAl_0.5_Si_0.5_H_*x*_, was seen at 340 °C. After heating to 480 °C, LaH_3_ peaks emerged. At 560 °C, the sample corresponded to a mixture of LaAlH_6_, LaH_3_, and Si. Figure S6 shows the Rietveld fit of the recovered sample. The lattice parameter of so obtained LaAlH_6_ is virtually identical to the one obtained from LaAl hydrogenations, cf. Table S1, indicating that Si cannot substitute for Al in LaAlH_6_.

**Figure 5:**
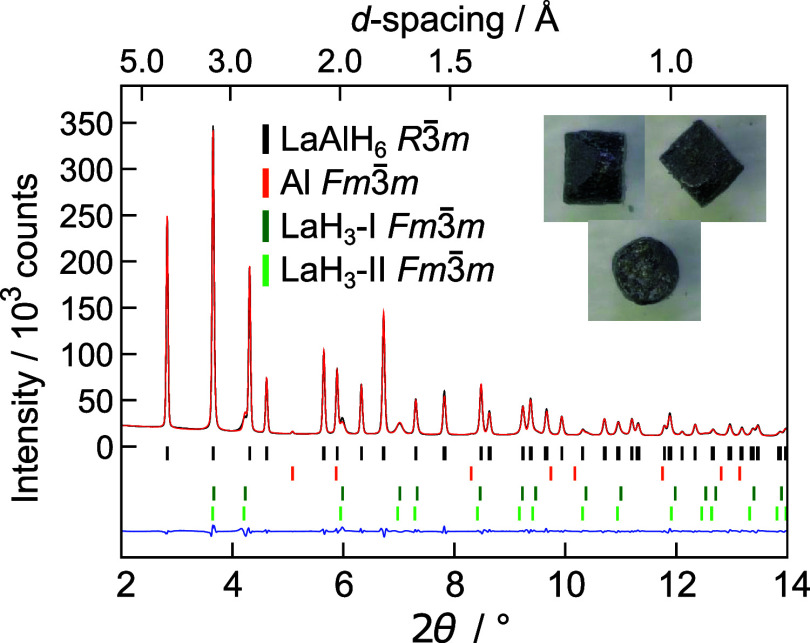
Rietveld fit to the synchrotron PXRD pattern (λ = 0.20734 Å, ambient conditions) of the product from the hydrogenation of LaAl at 10.2–12.1 GPa and 630 °C (cf. Figure 4). The inset shows photographs of the recovered sample (OD ∼ 1 mm). Lanthanum hydride reflections display an asymmetric peak shape. Refinement using two *Fm*3̅*m* structures resulted in a better fit, which could be attributed to a variable H content. Still, the phases are termed LaH_3_–I and LaH_3_–II for consistency. LaAlH_6_ *R*3̅*m* ^52^ (black): 85.2(3) wt %, Al *Fm*3̅*m* (orange): 1.6(1) wt %, LaH_3_–I *Fm*3̅*m* ^36^ (dark green): 8.7(1) wt %, LaH_3_–II *Fm*3̅*m* ^36^ (light green): 4.5(2) wt %. Lattice parameters LaAlH_6_ *R*3̅*m*: *a* = 6.51412(4) Å, *c* = 6.32682(6) Å, *V* = 232.502(3) Å^3^ (*R*_bragg_ = 1.13%, *R*_f_ = 0.975%), LaH_3_–I: *a* = 5.6231(2) Å, LaH_3_–II: *a* = 5.656(3) Å. *R*_p_ = 2.20%, *R*_wp_ = 3.09%, *R*_exp_ = 0.67%.

#### 3.2.3 LaSi

High pressure hydrogenations of LaSi were conducted at 9 and 20 GPa. The evolution of EDXRD patterns from the 9 GPa experiment upon heating is shown in Figure S7. At around 260 °C, after hydrogen release, reflections appeared that indicated the formation of a mixture of *Cmcm* and *Pnma* LaSiH. This would be similar to the outcome of earlier reported hydrogenations of LaSi at low (near ambient) pressure.^61^ The Le Bail analysis of the 345 °C pattern, shown in Figure S8, provides a rather good fit. However, the *Cmcm* model gives a too large volume compared to that of the DFT calculated *Cmcm* LaSiH structure at the same pressure (cf. Figure S11). Thus, the extracted *Cmcm* phase may contain additional hydrogen or the reflections do not represent a phase mixture but an unknown (mono)­hydride. At 440 °C, the appearance of LaH_3_ reflections indicated onset of decomposition, which was completed at 470 °C. At the same time, a set of new reflections emerged, which could not be assigned or indexed. This phase (or phase mixture) is stable until 570 °C and seems to be recoverable at ambient pressure; see Figure S9.

At 20 GPa, the onset of hydrogenation was observed at 460 °C by the appearance of a set of broad reflections, see Figure 6 (note that the decomposition behavior of BH_3_NH_3_ at pressures above 10 GPa is not well studied, but presumably occurs at between 350–400 °C and only releasing 2 × H_2_). Rather simultaneously with the emergence of broad hydride phase reflections, the formation of LaH_3_ was noticeable, which indicates decomposition of the initial ternary hydride phase. At 495–500 °C and within 25 min dwell time, reflections from the LaSi precursor had vanished and hydride reflections grew more prominent. The LeBail fit of the 500 °C pattern after the dwell is shown in Figure S10. The hydride phase likely corresponds to the predicted *Pnma* LaSiH_2_. The DFT calculated unit cell volume for *Pnma* LaSiH_2_ at around 20 GPa (shown in Figure S11) would match rather closely the unit cell volume obtained from the LeBail fit of the 500 °C data. However, the quality of diffraction data is low and additionally obscured by the simultaneous presence of broad LaH_3_ peaks. At 530 °C, LaSiH_2_ decomposed completely to LaH_3_ and Si with a simple hexagonal structure (Si–V). Lattice parameters for Si–V could be extracted via LeBail fitting using GSAS-II (530 °C: *a* = 2.5503­(5) Å, *c* = 2.3755(5) Å, *V* = 13.380(4) Å^3^; 700 °C: *a* = 2.5609(4) Å, *c* = 2.3773(4) Å, *V* = 13.501(3) Å^3^). Melting of Si–V occurred between 700 and 800 °C, which is in agreement with the reported melting curve of Si (∼780 °C at 20 GPa).^67^ After the sample is cooled to RT, Si is invisible in the EDXRD patterns. This is attributed to the formation of large Si grains after crystallization from the melt, producing spotty diffraction rings, which cannot be detected by the point detector. LaH_3_ forms simultaneously with LaSiH_2_ and its reflections continued to grow after decomposition of LaSiH_2_. Some reflections display broadening, indicating a possible deviation from the cubic cell. Above 700 °C, they sharpen, suggesting a transition to cubic symmetry (at 800 °C: *a* ≈ 5.31 Å), which is retained on cooling to RT. This low symmetry distortion was not observed clearly in the lower pressure runs.

**Figure 6:**
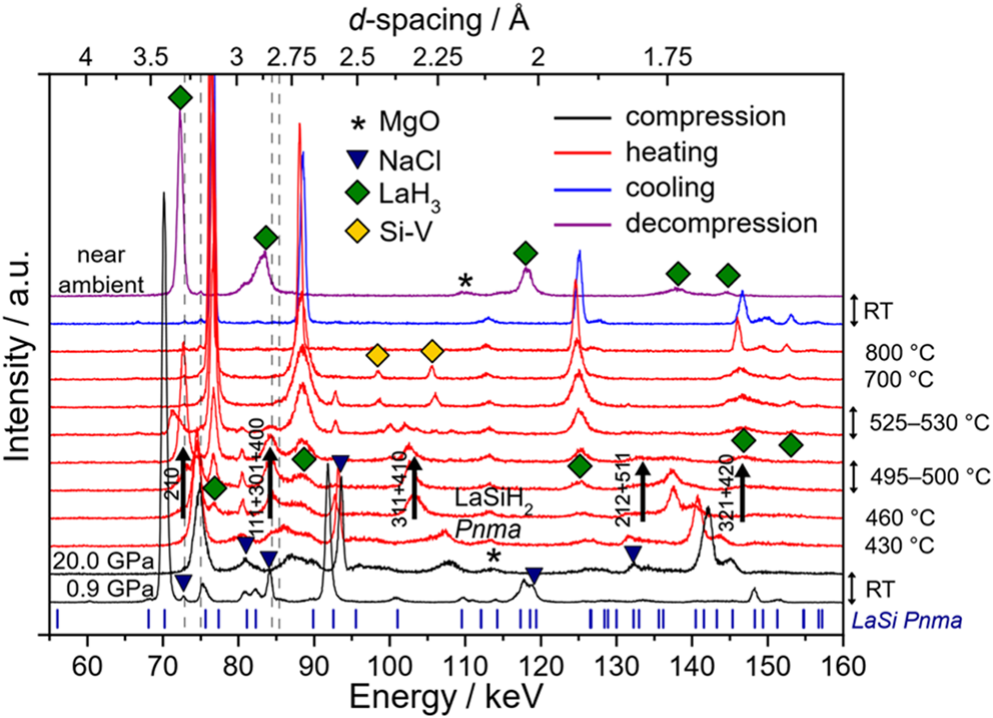
Hydrogenation of LaSi at 20 GPa. Diffraction patterns are shown for the starting and target pressure (black), during heating to 800 °C (red), after cooling to room temperature (blue), and after decompression (purple). Secondary Pb fluorescence peaks (from the detector shielding) are marked as dashed gray vertical lines. Green and yellow diamonds mark LaH_3_ and hexagonal Si–V. Blue triangles mark NaCl reflections from the sample capsule and asterisks mark MgO from the pressure cell assembly. The formation of the hydride phase at 460–500 °C, tentatively assigned as *Pnma* LaSiH_2_, is marked by black arrows. The origin of the peak at 137.5 keV, appearing at 460 °C and vanishing during dwelling at 495–500 °C, has not been identified. It is likely connected to the beginning of hydrogen uptake by the educt.

Although the formation of LaSiH_2_ remains ambiguous and there is no evidence of LaSiH_7_ in our experiment, we stress that CSP clearly indicates the stability of higher hydrides in La–Si–H at 20 GPa. In addition, phonon calculations show dynamic stability of LaSiH_2_ and LaSiH_7_ at 20 GPa (Figure S13) and also at ambient pressure (Figure S12), which strengthens the feasibility of their synthesis and, even more, their potential recovery at ambient pressure (although the kinetic stability of LaSiH_2_ and LaSiH_7_ at ambient pressure is uncertain). However, CSP is based on zero Kelvin calculations, whereas synthesis will require elevated temperatures for overcoming kinetic barriers. Assessing altered thermodynamic stability at finite temperature and kinetics for both formation and decomposition of hydride products, which often have different pathways, is computationally very expensive. The rather simultaneous formation of hydride and decomposition into LaH_3_ and Si suggests that required synthesis/reaction temperatures are comparable to the decomposition temperature at 20 GPa and, consequently, higher pressures and/or a higher H_2_ fluid activity would be needed. In this respect, we need to point out that the amount of H_2_ (×8H) employed for this experiment may not have been sufficient for hydridosilicate formation since BH_3_NH_3_ decomposition at 20 GPa (most likely) arrests at a polymeric “BHNH”. Against this background, a closer look at the compounds LaSiH*_n_* is warranted.

### 3.3 The (LaSi)_1–*x*_H_*x*_ Pseudobinary Line

Figure 7 depicts the convex hulls of the pseudobinary line (LaSi–H_2_) at 0 and 20 GPa, which are extracted from the data shown in Figure 2. These hulls reflect the stability of LaSiH*_n_* with respect to the reactants LaSi and H_2_ or, alternatively, mirror the energetics of the formation reaction. With respect to the ternary hull, LaSiH_6_ is more stable but still remains above the pseudobinary convex hull (by 15 meV/f.u.), which may be altered at a finite temperature. At ambient pressure, LaSiH_2_, LaSiH_6_, and LaSiH_7_ appear rather unstable, by about 75, 150, and 100 meV/f.u., respectively (with respect to LaSiH and H_2_). Yet these phases may be kinetically stable.

**Figure 7:**
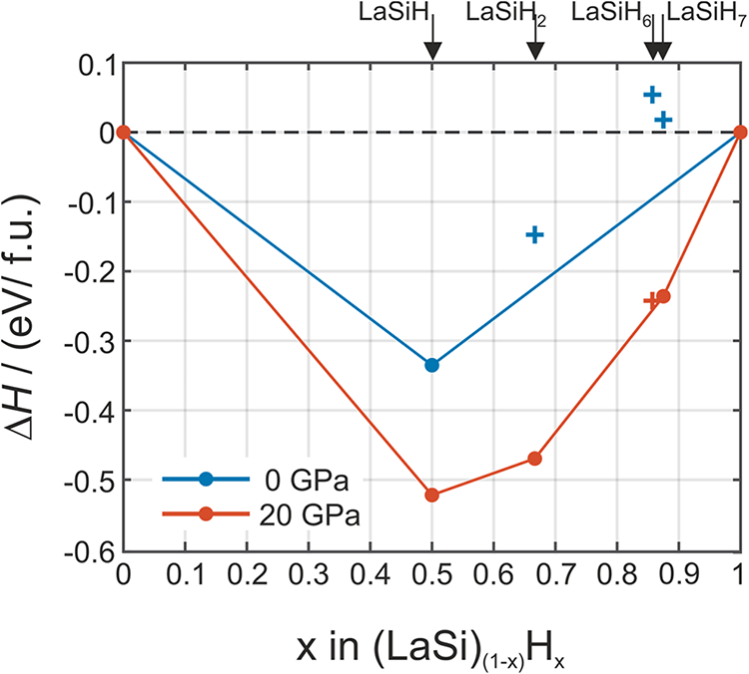
Convex hulls for the pseudobinary LaSi – H_2_ line at 0 and 20 GPa (cf. Figure 3). Stable phases at 20 GPa (red circles) are *Cmcm* LaSiH, *Pnma* LaSiH_2_, and *P*1 LaSiH_7_. *R*3̅ LaSiH_6_ is above the hull by only 15 meV/atom (red cross). At 0 GPa, *Cmcm* LaSiH remains thermodynamically stable, whereas the higher hydrides (blue crosses) only retain dynamic stability, see phonon dispersions provided as Supporting Information, Figures S12 and S13, respectively.

Figure 8 compares the electronic structures, band structures, and density of states (DOS) of LaSiH, LaSiH_2_, LaSiH_6_, and LaSiH_7_ at their calculated equilibrium volumes, i.e., at 0 GPa. LaSiH appears metallic, although it formally corresponds to a charge-balanced Zintl phase La^3+^[Si]^2–^(H^–^), Figure 8a. The Fermi level is located in a pronounced pseudo gap in the DOS. Noticeably, a large contribution of La states to occupied bands, in agreement with an earlier analysis of the electronic structure of LaSiH.^61^ According to the Zintl concept, LaSiH_2_ is imbalanced, La^3+^[SiH]^−^(H^–^)­e. Its DOS (Figure 8b) has some resemblance to LaSiH, but at the same time, it deviates considerably from a rigid band behavior. The lowest lying bands, with mostly Si-*s* contribution, are detached and confined in the energy range −11 to −8 eV. The pseudo gap seen for LaSiH is maintained and located at around −2 eV.

**Figure 8:**
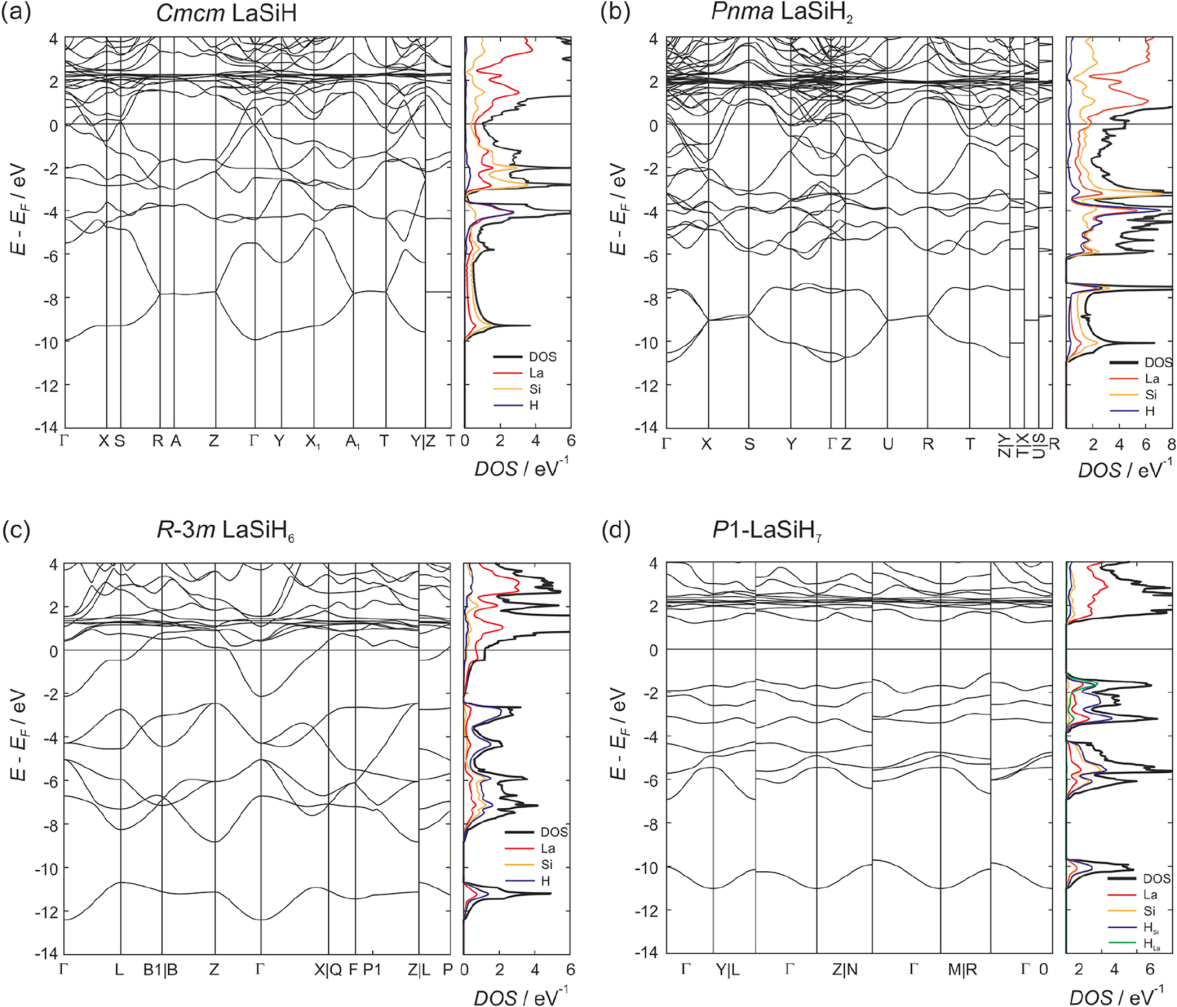
Electronic band structure and density of states (DOS) of *Cmcm* LaSiH (a), *Pnma* LaSiH_2_ (b), *R*3̅*m* LaSiH_6_ (c), and *P*1 LaSiH_7_ (d) at their calculated equilibrium volumes (zero pressure). The DOS is partitioned into contributions of atoms. The H atoms of LaSiH_7_ are divided between H atoms coordinated to Si (6) and the H atom exclusively coordinated to La (green line).

The electronic structures of *R*3̅*m* LaSiH_6_ and *P*1 LaSiH_7_ are rather easy to interpret. Occupied states relate to bonding and nonbonding molecular orbitals of the hypervalent SiH_6_^2–^ ion, six per f.u.^64^ For charge-imbalanced LaSiH_6_ (La^3+^[SiH_6_]^2–^e), these Si–H based bands are separated by a small gap from the conduction band (which has a large La contribution) and which hosts the additional electron, Figure 8c. For charge-balanced LaSiH_7_, La^3+^[SiH_6_]^2–^(H^–^), there is a sizable (≈3 eV) band gap at the Fermi level. The states of the hydridic H are largely confined in the narrow range between −4 and −2 eV, where also the nonbonding (e_g_-type) states of the SiH_6_^2–^ moiety are located, as shown in Figure 8d. The evolution of the electronic structure from the lower interstitial hydrides LaSiH and LaSiH_2_ to the higher hydridosilicates is also clearly reflected in the Bader charge analysis (Table S7). The charge on Si changes from negative to positive, accompanied by a pronounced reduction in its Bader volume. In contrast, the charge on La increases slightly from approximately +1.5 to +1.8, while the hydrogen atoms carry charges in the range of −0.55 to −0.67.

Since LaSiH, LaSiH_2_, and LaSiH_6_ represent metals, it will be interesting to assess their superconducting properties. Figures S12 and S13 (showing the calculated phonon dispersion relations) also include phonon densities of states *F*(ω), Eliashberg electron–phonon coupling functions α^2^ *F*(ω), and the cumulative electron–phonon coupling constants λ­(ω). Values of the calculated superconductor parameters are presented in Table 2. LaSiH attains very low critical temperatures, below 1 K, whereas LaSiH_2_ has a *T*_c_ in the range 9–11 K, slightly increasing with pressure, and LaSiH_6_ has a *T*_c_ of 6 K at 0 GPa and about 9 K at 20 GPa, indicating a moderately strong electron-phonon coupling for both materials.

**Table 2:** Calculated Superconductor Parameters for *Cmcm* LaSiH, *Pnma* LaSiH_2_, and *R*3̅*m* LaSiH_6_. (λ = Electron-Phonon Coupling Constant, ω_log_ = Logarithmically Averaged Phonon Frequency, DOS­(*E*_F_) = Number of States at the Fermi Level)

compound, *p*	λ	ω_log_/meV	DOS(*E*_F_)/eV^–1^	*T*_*c*_/K
*Cmcm* LaSiH, 0 GPa	0.28	27.0	1.07	0.8
*Cmcm* LaSiH, 20 GPa	0.21	34.8	0.87	0.3
*Pnma* LaSiH_2_, 0 GPa	0.65	30.4	4.47	9.4
*Pnma* LaSiH_2_, 20 GPa	0.61	37.9	4.11	11.1
*R*3̅*m* LaSiH_6_, 0 GPa	0.54	30.1	1.21	5.0
*R*3̅*m* LaSiH_6_, 20 GPa	0.54	45.0	1.10	7.3

## 4 Conclusions

The ternary systems La–Al–H and La–Si–H were investigated at pressures up to 20 GPa by computational structure prediction and *in situ* synchrotron diffraction studies. In the La–Al–H system, LaAlH_6_ is the only stable ternary compound for 0 and 20 GPa predicted by CSP and in the experiments conducted at 2 and up to 12 GPa. While already reported with the *R*3̅*m* structure (BaSiF_6_ type), we find that *R*3̅ is slightly more stable at ambient conditions and increasingly more stable with pressure.

More variability was found in the La–Si–H system. At ambient pressure, only LaSiH is thermodynamically stable, with its *Cmcm* structure slightly more favored than the *Pnma*. At 20 GPa, LaSiH_2_ (*Pnma*) and the semiconducting hydridosilicate LaSiH_7_ are additionally stable. LaSiH_6_ was found to be potentially stable at finite *T* or as a metastable phase. Among the metals LaSiH, LaSiH_2_, and LaSiH_6_, the latter two are superconducting with a moderately strong e-ph coupling. Our calculations suggest that LaSiH_2_ has a *T*_c_ of 9 K at 0 GPa and 11 K at 20 GPa, and LaSiH_6_ has a *T*_c_ of 5 K at 0 GPa and 7 K at 20 GPa. While LaSiH_6_ and LaSiH_7_ could not be observed experimentally, indications for a possible LaSiH_2_ formation were present but could not be unambiguously confirmed. Simultaneous decomposition into LaH_3_ and Si occurred, suggesting that pressures above 20 GPa and/or higher H_2_ concentrations are necessary.

## Supplementary Material



## Notes

Description of LaAl, LaSi, and LaAl_0.5_Si_0.5_ precursor characterization; DFT calculated enthalpy difference between *R*3̅*m* and *R*3̅ LaAlH_6_ as a function of pressure; DFT calculated enthalpy difference between *Cmcm* LaSiH and *Pnma* LaSiH as a function of pressure; *in situ* EDXRD patterns obtained during the hydrogenation of LaAl at 2 GPa; *in situ* EDXRD patterns obtained during the hydrogenation of LaAl_0.5_Si_0.5_ at 11 GPa; compilation of LaAlH_6_ lattice parameters at various *p*, *T* conditions; *in situ* EDXRD patterns obtained during the hydrogenation of LaSi at 9 GPa; *ex situ* evaluation of product from the LaSi-H_2_ experiment at 9 GPa; Le Bail fits for La_2_AlSi-H_2_, 11 GPa, 305 °C; LaSi-H_2_, 9 GPa, 345 °C; LaSi-H_2_, 20 GPa, 500 °C; DFT calculated *V/Z* vs pressure; phonon dispersion and electron–phonon coupling calculations for *Cmcm* LaSiH, *Pnma* LaSiH_2_, *R*3̅*m* LaSiH_6_, *P*1 LaSiH_7_ at 0 and 20 GPa; DFT calculated structure data for LaAlH_6_ and LaSiH*_n_* (*n* = 1,2,6,7); Bader charges and volumes for LaSiH*_n_* (*n* = 1,2,6,7) (PDF)
