# Supplementary material for: Investigation of the La–Al–H and La–Si–H Systems at High Pressures
Source: Inorg Chem. 2026 Jan 22;65(4):2336–46. doi: 10.1021/acs.inorgchem.5c05140 (PMC12869500; doi:10.1021/acs.inorgchem.5c05140)
Supplement: Supplementary file 1 [file ic5c05140_si_001.pdf]

SUPPORTING INFORMATION

Investigation of the La-Al-H and La-Si-H systems at high pressures

Doreen C. Beyer,[1] Pedro Nunes Ferreira,[2,3] Roman Lucrezi,[4] Luiz Tadeu Fernandes Eleno,[2] Holger Kohlmann,[1] Christoph Heil,[3] Michael Sannemo Targama,[4] Volodymyr Baran,[5] Shrikant Bhat,[5] Robert Farla,[5] Kristina Spektor,[5] and Ulrich Häussermann[4,*]

[1]*Leipzig University, Faculty of Chemistry, Institute of Inorganic Chemistry and Crystallography, Johannisallee 29, D-04103 Leipzig, Germany*

[2]*DEMAR Escola de Engenharia de Lorena, Universidade de São Paulo, 12612-550, Lorena, Brazil*

[3]*Institute of Theoretical and Computational Physics, Graz University of Technology, NAWI Graz, 8010 Graz, Austria*

[4]*Department of Chemistry, Stockholm University, 10691 Stockholm, Sweden*

[5]*Deutsches Elektronen-Synchrotron DESY, Notkestr. 85, 22607 Hamburg, Germany*

*email: Ulrich.Hausssermann@su.se

I. Precursor characterization

II. Supporting figures

I. Precursor characterization

All three precursors LaAl, $LaAl_{0.5}Si_{0.5}$ and LaSi were prepared by arc melting stoichiometric mixtures of the elements (La, Chempur, 99.9 %; Si, ABCR GmbH, 99.9999 %; Al shots, ABCR GmbH, 99.999 %). According to the La–Si phase diagram reported by M. V. Bulanova et al. LaSi forms congruently at 1620 °C.[1] LaSi was obtained as a virtually phase pure sample.

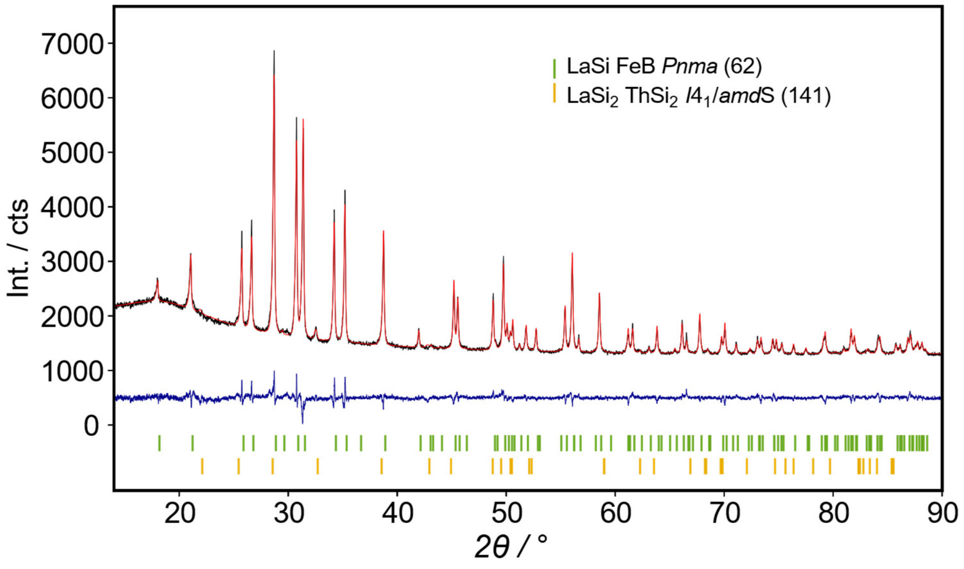

Rietveld fit to the measured powder X-ray diffraction (PXRD) patterns (Cu Kα radiation) of the utilized LaSi precursor obtained from arc-melting. LaSi (green):[2] 97.4 wt%, $LaSi_2$ (yellow):[3] 2.6 wt%. R_{exp}: 2.44 %, R_{wp}: 1.98 %, R_p: 1.43 %, GOF: 0.81. Rietveld analysis was performed with Topas.[4,5]

In contrast, LaAl and $LaAl_{0.5}Si_{0.5}$ form incongruently and precursor samples were afforded as phase mixtures. The binary La–Al phase diagram from S. H. Zhou et *al.*[6] appears incomplete since also a hexagonal phase La_5Al_4 has been reported.[7]

LaAl has a rather complex and unique orthorhombic crystal structure[8] whereas $LaAl_{0.5}Si_{0.5}$ adopts the prolific CrB structure type.[9] The LaAl and $LaAl_{0.5}Si_{0.5}$ precursor samples were characterized by ICP analysis, PXRD and SEM investigations. Results and details are presented below. Importantly, the overall composition of the samples corresponded closely to the nominal synthesis compositions.

ICP analysis of LaAl and $LaAl_{0.5}Si_{0.5}$ samples

(result of two determinations)

	at.%/wt.% La	wt.% La	at.% Al	wt.%Al	at.%Si	wt.%Si
**$LaAl_{0.5}Si_{0.5}$**	50.18	81.92	25.01	7.93	24.81	8.19
	50.17	81.88	25.08	7.95	24.76	8.17
**LaAl**	50.38	83.41	49.62	15.96		
	50.33	83.37	49.67	15.98		

EDS analysis and mapping of LaAl and $LaAl_{0.5}Si_{0.5}$ precursor samples

Ingots obtained from arc melting were fragmented to smaller pieces in an agate mortar by a few gentle taps with a pestle. A fragment was then picked out and glued to an Al stub using hot-melt glue for subsequent cross-section polishing (CP). A pre-polishing step was performed using a surfacing jig on abrasive paper (4000P grit, ~5 µm) to get a flat initial surface before the CP. Ar ion beam CP was performed with a JEOL SM-09010 cross-section polisher, using a 5.3 kV beam voltage and polishing for 20 h. To avoid any potential oxidation or surface degradation from contact with air, samples were stored in an Ar-filled glove box when they were not being measured.

Scanning electron microscopy (SEM) analyses were performed on a Hitachi TM3000 table-top scanning electron microscope equipped with a thermionic electron gun, a back-scattered electron (BSE) annular detector (with 4 segments) and a detector for energy dispersive x-ray spectroscopy (EDS) analyses. Samples were measured using a 15 kV acceleration voltage.

EDS spectra were collected for a larger region first to ascertain that the average composition was near to what was weighed in, then individual spots were analyzed at a higher magnification for each of the discernable phases. This was done by performing a region scan with a 10 min scan time. Then, in said maps, individual spot regions were inspected, and the elemental compositions were calculated using the built-in EDS software (Quantax70).

Results are compiled in the two following figures. For the LaAl sample BSE images and EDS analysis suggest that besides the main phase LaAl there are also phases with compositions $LaAl_2$, La_5Al_4 and La_2Al. The overall EDS composition agrees well with the ICP result (ca. 50 at.% La and Al). For the $LaAl_{0.5}Si_{0.5}$ sample the main phase appears slightly rich in Si ($LaAl_{0.45}Si_{0.55}$). The additional phases are Al-rich and may derive from $LaAl_2$ and La_5Al_4 with some (up to 20 %) Al substituted by Si ($La(Al_{0.88}Si_{0.12})_2$ and $La_5(Al_{0.81}Si_{0.19})_4$, respectively). Again, the overall composition determined by EDS agrees well with the ICP result (ca 50 at.% La and 25 at.% Al and Si).

LaAl

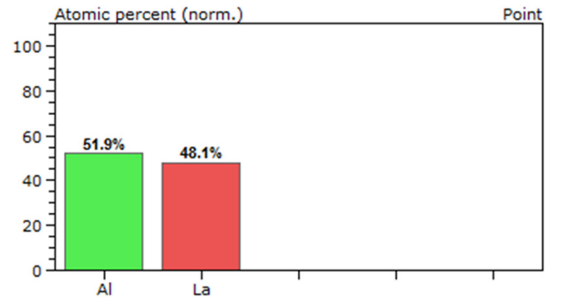

Element	Atomic No	Series	at. %	Error
Al	13	K	51.93	0.8
La	57	L	48.07	2.2

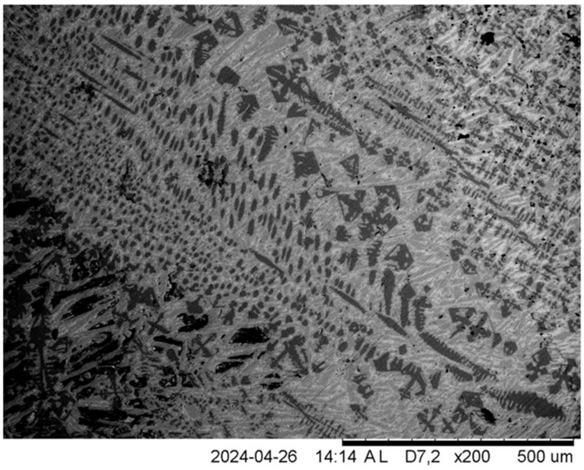

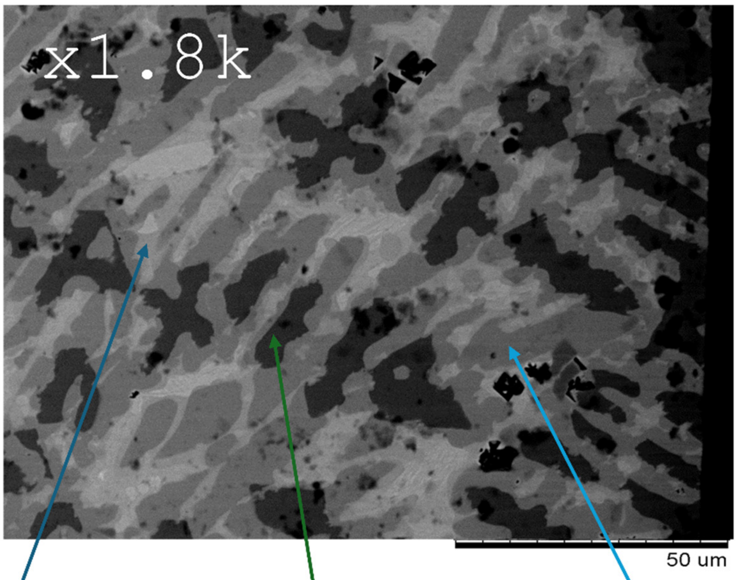

	Bright spots		Dark Spots		Medium spots (main phase)	
	La	Al	La	Al	La	Al
	57.3	42.7	31.2	68.8	40.7	59.3
	66.9	33.1	31	69	44.7	55.3
	57.2	42.8	35.5	64.9	49.4	50.6
	57.9	42.1	35.9	64.1	49.2	50.8
	63.9	36.1	34	66	54.9	45.1
	61.1	38.9	33.7	66.3	45.2	54.8
	57.2	42.8	34.7	65.3	47.3	52.7
	58.1	41.9	31.4	68.6	47.8	52.2
Average	**60.0**	**40.1**	**33.4**	**66.6**	**47.4**	**52.6**
Std. Dev.	**3.68**	**3.68**	**1.98**	**1.92**	**4.15**	**4.15**
	La_5Al_4 + La_2Al		$LaAl_2$		LaAl	

$LaAl_{0.5}Si_{0.5}$

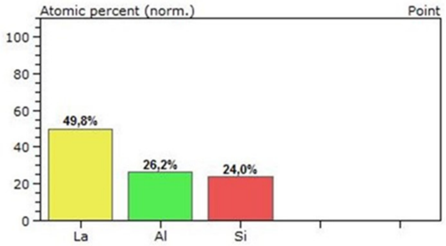

Element	Atomic No.	Series	at. %	Error
La	57	L	49.81	2.3
Al	13	K	26.24	0.4
Si	14	K	23.95	0.3

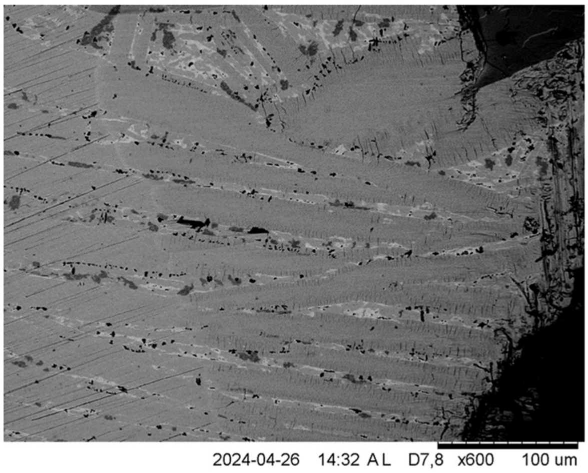

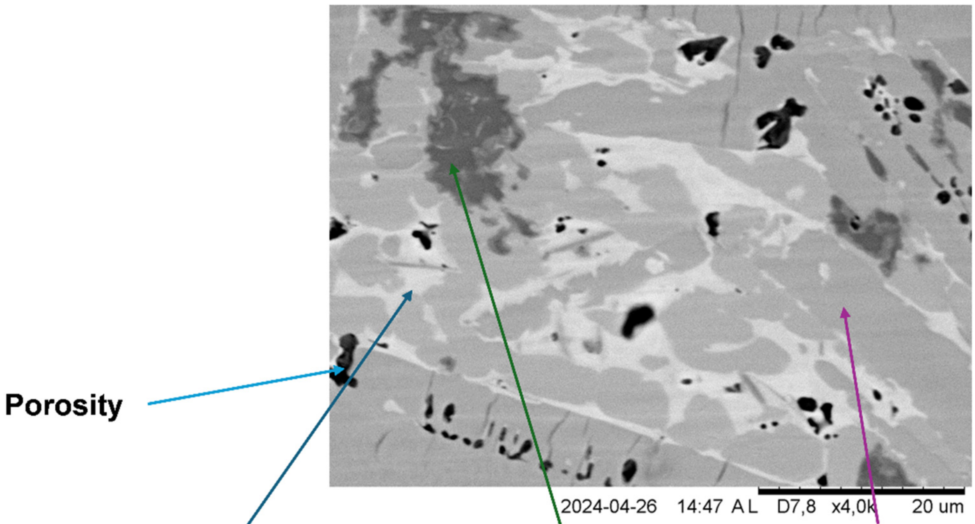

	Bright Spots			Darker Spots			Medium dark spots (Main Phase)		
	La	Si	Al	La	Si	Al	La	Si	Al
	65.0	5.9	29.1	39.0	5.5	55.4	52	29.1	18.9
	52.5	7.4	40.1	36.8	9.6	53.6	48.8	29	22.2
	53.8	4.5	41.7	35.1	8.3	56.6	45.2	31.8	23.0
	60.5	8.0	31.5				46.4	29.1	24.5
							45.5	28.9	25.6
							45.9	27.7	26.3
	59.2	4.8	36.1				47.6	27	25.4
	51.8	17.9	30.3				47.5	28.7	23.9
Average	**57.1**	**8.1**	**34.8**	**37.0**	**7.8**	**55.2**	**47.4**	**28.9**	**23.7**
Std. Dev.	**5.26**	**5.00**	**5.31**	**1.96**	**2.10**	**1.51**	**2.23**	**1.39**	**2.38**

PXRD analysis of LaAl and $LaAl_{0.5}Si_{0.5}$ samples

Fragments of ingots obtained from arc melting were ground into fine powders. Powder X-ray diffraction (PXRD) patterns were collected on a Panalytical X'Pert PRO diffractometer operated with Cu Kα radiation and in θ-2θ diffraction geometry. Powder samples were applied to a zero diffraction plate and diffraction patterns measured in a 2θ range 13–90°. The diffraction patterns were evaluated with PDIndexer (ver.4.412)[10] and are shown below. The quality and multi-phase nature of the patterns rendered Rietveld refinements unsuccessful.

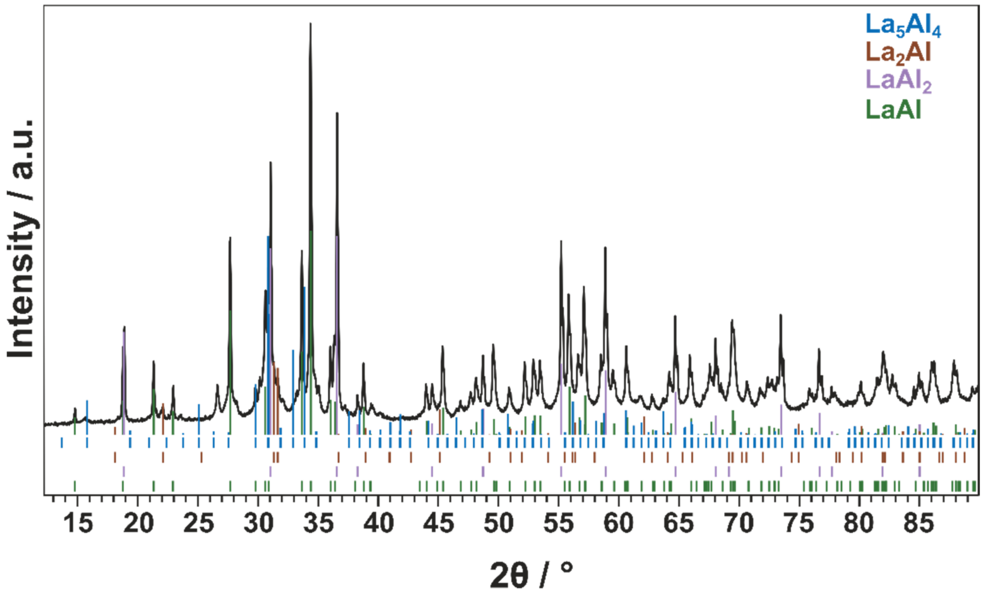

PXRD pattern of the LaAl precursor sample (Cu Kα radiation); LaAl[8] (green), $LaAl_2$[11] (pink) La_2Al[12](brown), and La_5Al_4[7] (blue)

The PXRD pattern of the LaAl precursor revealed unambiguously the presence of the phases LaAl and $LaAl_2$, in accordance with the EDS analysis. On the other hand, the presence of hexagonal La_5Al_4 cannot be concluded with certainty. EDS analysis suggested further the existence of a yet unreported phase La_2Al. Unassigned reflections in the PXRD patterns match to a hexagonal cell with $a \approx 5.655$ Å and $c \approx 7.040$ Å, which could correspond to La_2Al with a Ni_2In structure. La_2In has been reported with the Ni_2In structure ($a = 5.636$ Å, $c = 7.065$ Å).[12] Still unaccounted reflections hint at more unidentified phases in the LaAl precursor sample.

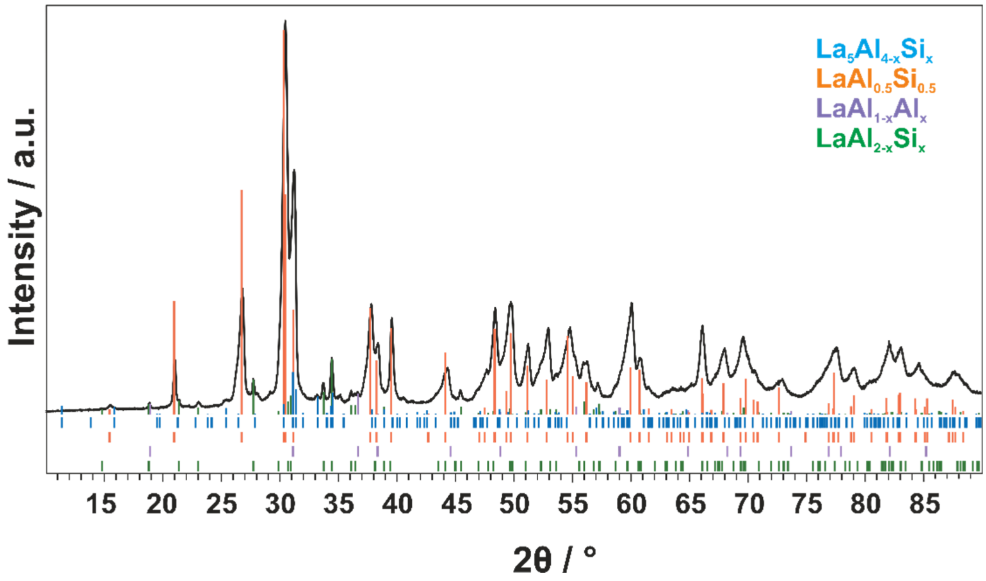

PXRD pattern of the $LaAl_{0.5}Si_{0.5}$ precursor sample (Cu Kα radiation); $LaAl_{0.5}Si_{0.5}$[9] (orange), $LaAl_{1-x}Si_x$ (green), and $LaAl_{2-x}Si_x$ (pink).

The PXRD pattern of the $LaAl_{0.5}Si_{0.5}$ precursor sample confirmed the presence of orthorhombic $LaAl_{0.5}Si_{0.5}$ (CrB structure, $a \approx 4.562$ Å, $b \approx 11.56$ Å, $c \approx 4.161$ Å)[9] as the majority phase. $LaAl_2$ and LaAl (with some Si incorporated) appear to be side phases (although the latter was not suggested by EDS analysis). Similar to the LaAl precursor sample, it is not clear whether a hexagonal phase $La_5Al_{4-x}Si_x$ exists and is present.

II. Supporting figures

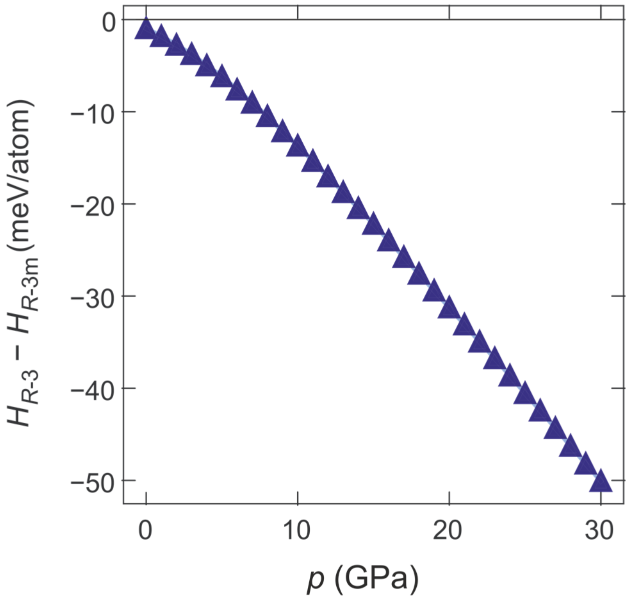

Figure S1. Enthalpy difference of *R*-3*m* and *R*-3 $LaAlH_6$ with increasing pressure.

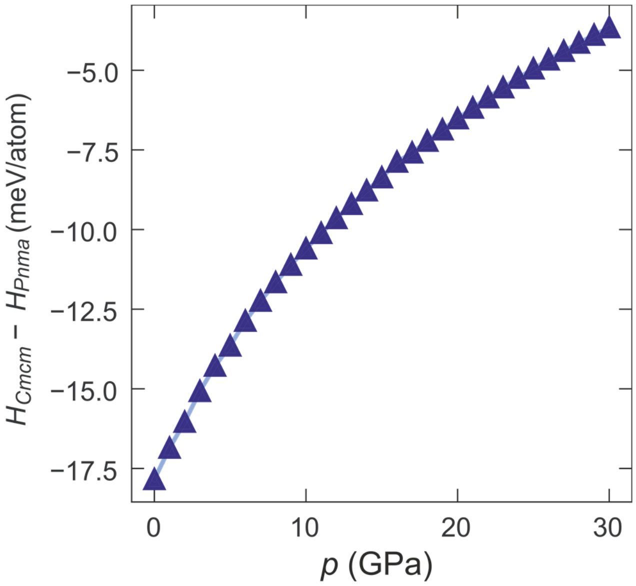

Figure S2. Enthalpy difference of *Cmcm* and *Pnma* LaSiH with increasing pressure.

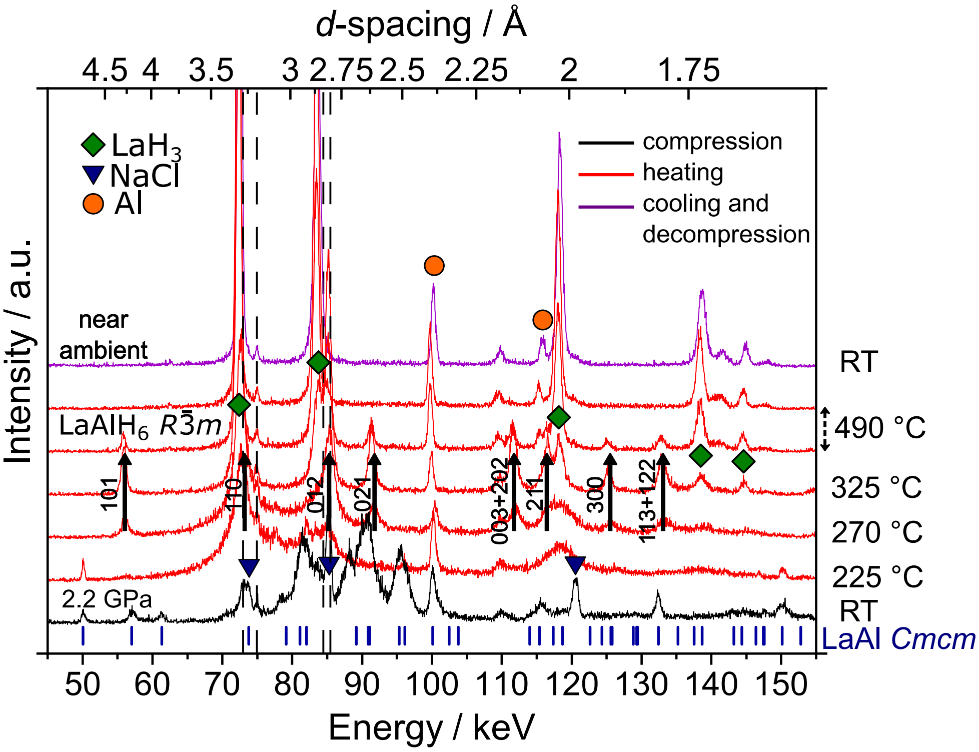

Figure S3. Hydrogenation of LaAl at around 2 GPa. Diffraction patterns at target pressure (2.2 GPa), heating to 490 °C, and after decompression at room temperature are shown in black, red, and purple, respectively. Secondary Pb fluorescence peaks (from the detector shielding) are marked as dashed gray vertical lines, blue triangles mark NaCl reflections from the sample capsule. The formation of $LaAlH_6$ starting at 270 °C is marked by arrows and the decomposition products LaH_3 and Al are marked by green diamonds and orange circles, respectively.

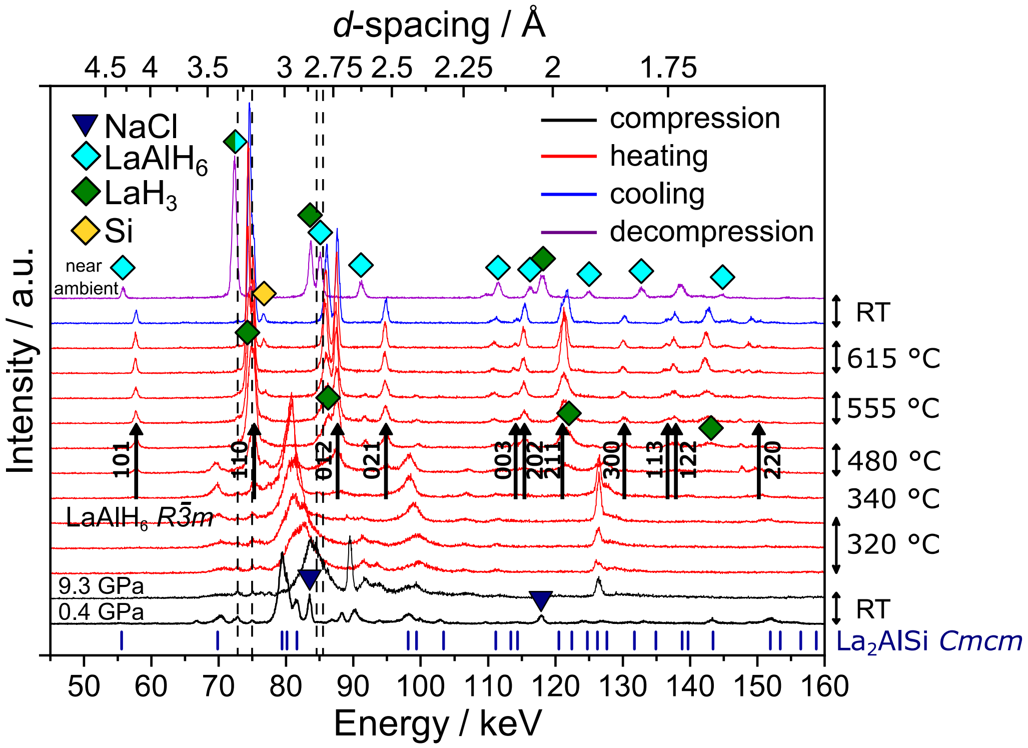

Figure S4. Hydrogenation of $LaAl_{0.5}Si_{0.5}$ at around 9 GPa. Diffraction patterns are shown for starting and target pressure (black), during heating to 615 °C (red), after cooling to room temperature (blue) and after decompression (purple). Secondary Pb fluorescence peaks (from the detector shielding) are marked as dashed gray vertical lines, blue triangles mark NaCl reflections from the sample capsule. The formation of $LaAlH_6$ (marked by arrows) is observed at 340 °C. After heating to 480 °C LaH_3 peaks emerge. Reflections from $LaAlH_6$, LaH_3 and α-Si are marked with blue, green, and yellow diamonds, respectively. While heating to 340 °C the formation of a potentially mixed intermediate hydride $LaAl_{0.5}Si_{0.5}H$ (see fig. S5) was visible, which vanished at 480 °C with the formation of $LaAlH_6$.

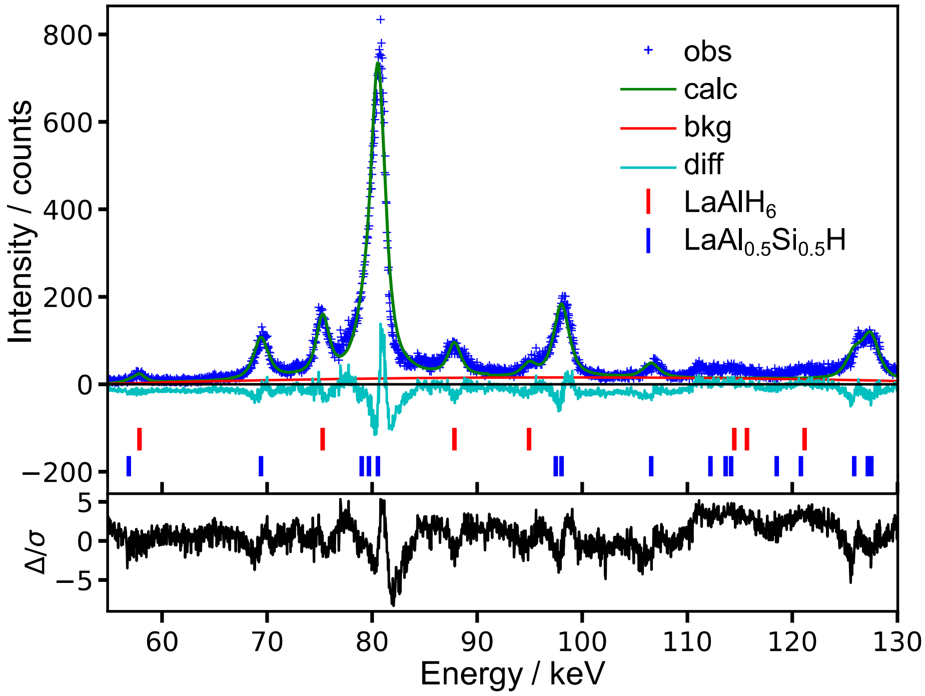

Figure S5. Hydrogenation of $LaAl_{0.5}Si_{0.5}$ at around 9 GPa (cf. Fig. S4). LeBail fit to the diffraction pattern at 390 °C. Lattice parameters of *Cmcm* "$LaAl_{0.5}Si_{0.5}H_x$": a = 4.393(3) Å, b = 11.853(7) Å, c = 4.101(2) Å, V = 213.5(1) Å^3. For the fit, a calculated LaSiH *Cmcm* model was used, while a mixed valence was assumed due to the absence of Si or other side phases containing Si. Additionally in the La–Al 10 GPa (see main text, Fig. 4) and La–Si 9 GPa experiment, while the formation of intermediate phases is observed, reflection positions indicate different compounds.

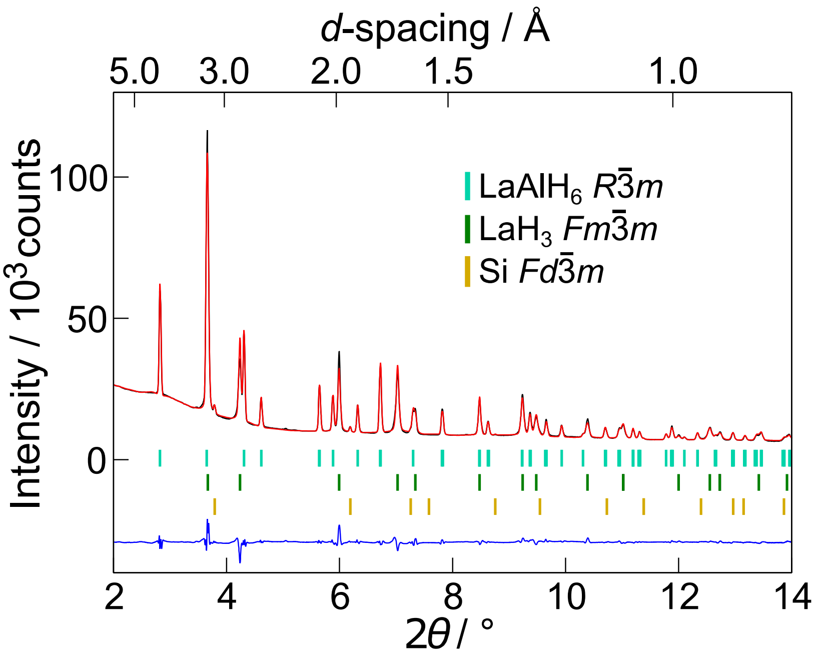

Figure S6. Rietveld fit to the synchrotron PXRD pattern (λ = 0.20734 Å, ambient conditions) of the product from the hydrogenation of $LaAl_{0.5}Si_{0.5}$ at 9.3 GPa and 615 °C (cf. Fig. S5). Lanthanum hydride reflections display an asymmetric peak shape. However, compared to Fig. 5 (main text), the pattern was satisfactorily refined by assuming a single phase. $LaAlH_6$ *R*-3*m* (turquoise): 49.1(6) wt%, LaH_3 *Fm*-3*m* (green): 46.5(5) wt%, Si *Fd*-3*m* (yellow): 4.4(3) wt%. Lattice parameters $LaAlH_6$ *R*-3*m*: a = 6.5154(3) Å, b = 6.5154(3) Å, c = 6.3247(3) Å (R_{bragg} = 4.07 %, R_f = 3.35 %). R_p = 2.26 %, R_{wp} = 3.49 %, R_{exp} = 0.89 %, χ^2 = 15.5.

Table S1. Compilation of $LaAlH_6$ lattice parameters at various *p*, *T* conditions. Comparison of the lattice parameters of the pure $LaAlH_6$ and the one from the Al/Si-mixture does not suggest any incorporation of Si.

**Experiment**	**Conditions**	***a* / Å**	***c* / Å**	***V* / Å³**
LaAl-H 10-12 GPa (*in situ*)	9.9 GPa, 450 °C	6.205(2)	6.119(3)	204.00(9)
	12.1 GPa, 465°C	6.136(1)	6.0966(9)	198.9(1)
	12.1 GPa, 630 °C	6.146(2)	6.110(1)	199.86(7)
	11.0 GPa	6.1409(8)	6.0921(5)	198.96(3)
	0.4 GPa	6.496(1)	6.3296(6)	231.28(4)
LaAl-H 10-12 GPa (*ex situ*)	Ambient	6.51412(4)	6.32681(6)	232.502(3)
$LaAl_{0.5}Si_{0.5}$-H 11 GPa (*ex situ*)	Ambient	6.5154(3)	6.3247(3)	232.51(2)
Literature[13]	Ambient	6.5272(4)	6.3212(7)	233.23

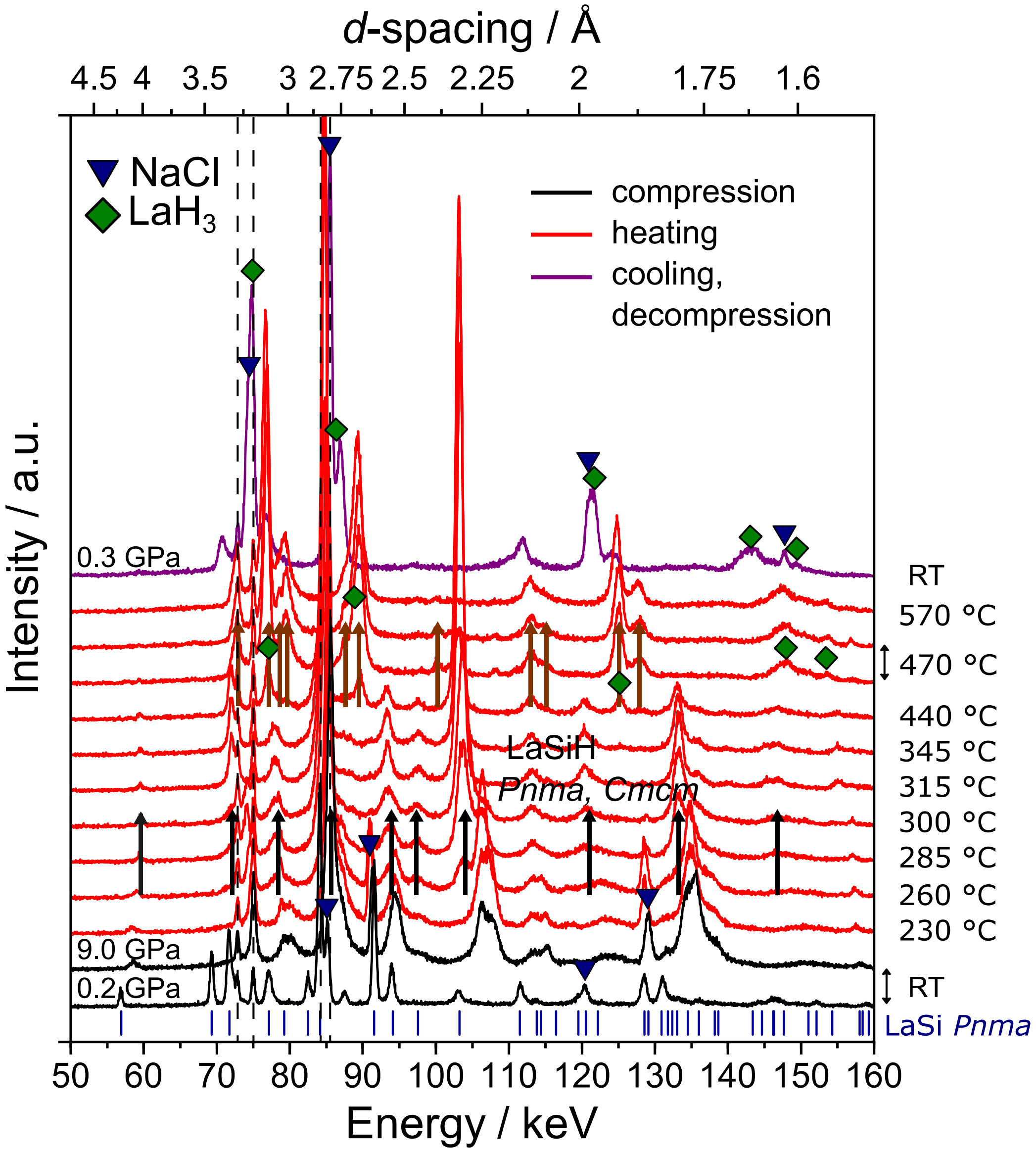

Figure S7. Hydrogenation of LaSi at 9 GPa. Diffraction patterns are shown for starting and target pressure (black), during heating to 570 °C (red), and after decompression (purple). The formation of interstitial hydride LaSiH (marked by arrows) is observed at 260 °C (cf. Fig. S8). After heating to 440 °C LaH_3 peaks emerge, together with at least one new unidentified phase (marked by brown arrows). Reflections from LaH_3 are marked with green diamonds.

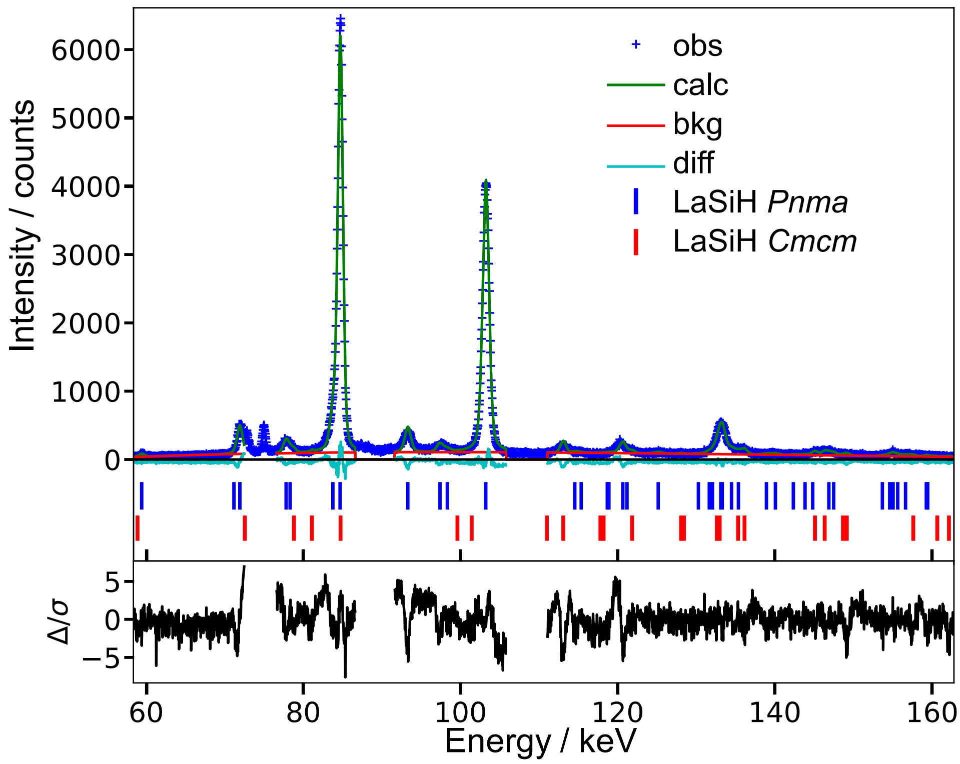

Figure S8. LeBail fit to the EDXRD pattern from the hydrogenation of LaSi at around 9 GPa and 345 °C. (cf. Fig. S7). LaSiH *Pnma*: a = 8.044(2) Å, b = 3.9690 (6) Å, c = 6.1067(6) Å, V = 194.97(3) Å^3. LaSiH *Cmcm*: a = 4.323(1) Å, b = 12.124 (2) Å, c = 3.916 (1) Å, V = 205.26(5) Å^3.

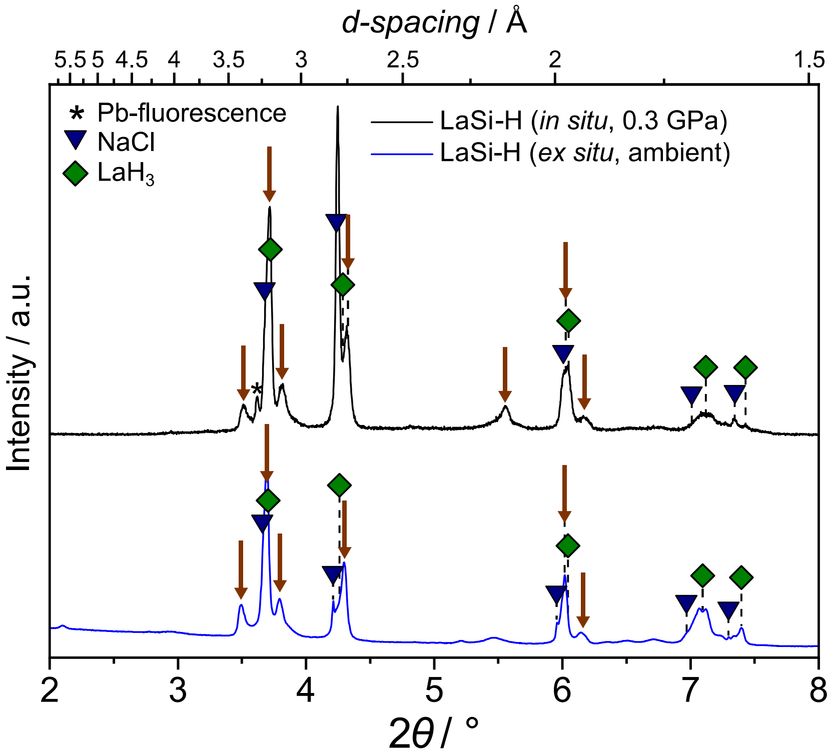

Figure S9. Room temperature PXRD patterns (λ ≈ 0.2073 Å) of the decompressed (0.3 GPa, top) and recovered La–Si–H sample (ambient pressure, bottom) after heating to 570 °C at ~9 GPa (cf. Fig. S7). NaCl and LaH_3 are marked with blue triangles, green diamonds. Unknown phase(s) are marked by brown arrows. Due to heavy peak overlap, broad reflections and the presence of at least one unidentified phase. Rietveld refinement was not possible.

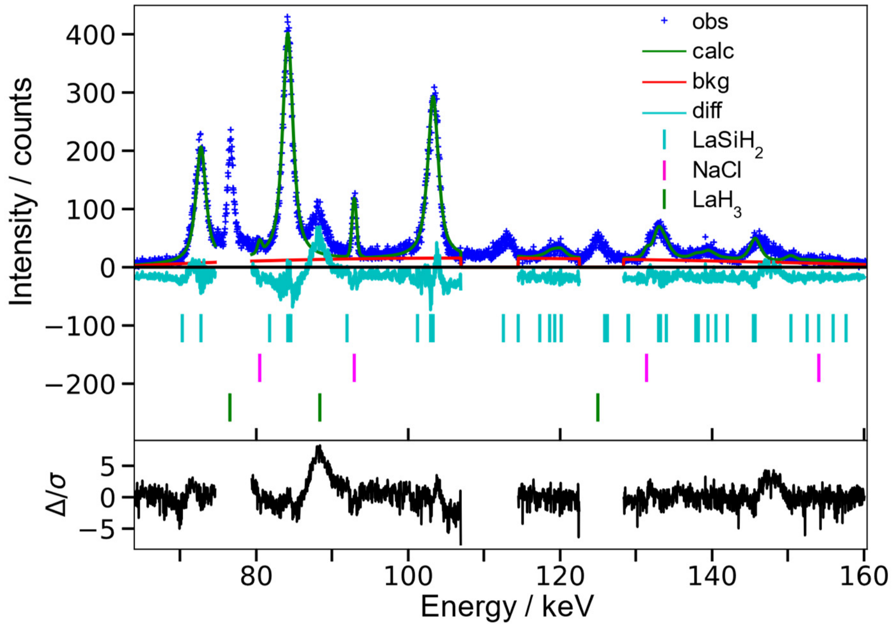

Figure S10. LeBail fit to the EDXRD pattern from the hydrogenation of LaSi at around 20 GPa and 500 °C. $LaSiH_2$ *Pnma* (turquoise): a = 11.127(2) Å, b = 3.952(1) Å, c = 4.156(2) Å, V = 182.8(1) Å^3. LaH_3 reflections are marked (green) but were not included in the refinement due to poor crystallinity and possible low symmetry distortion.

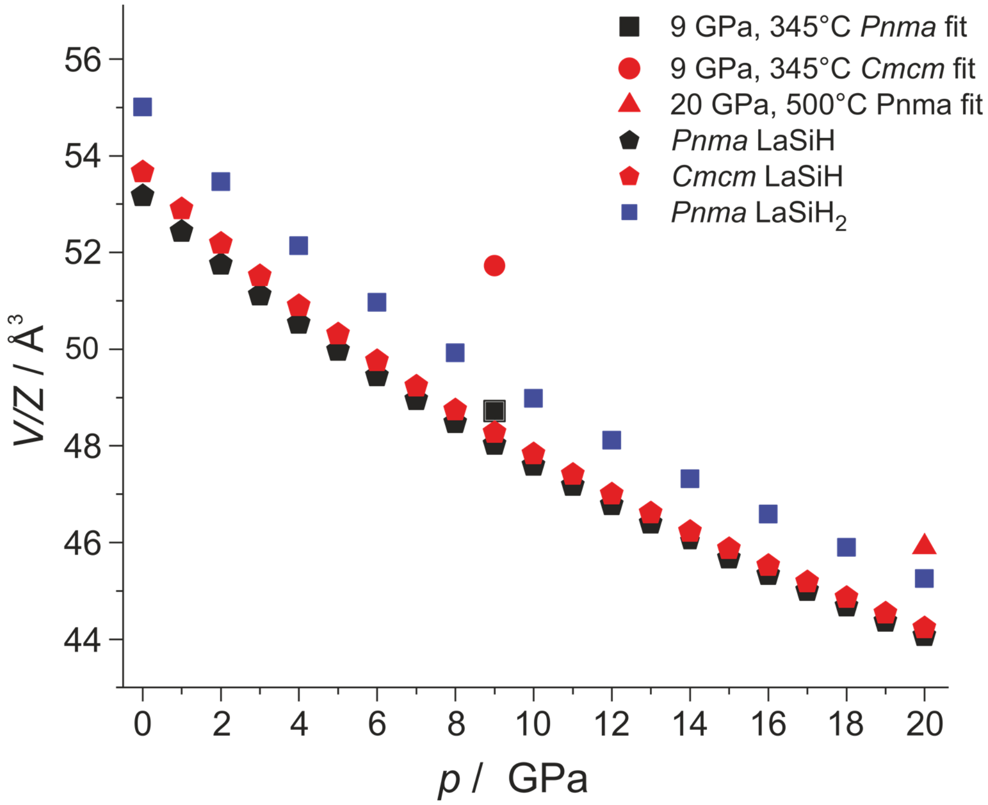

Figure S11: DFT calculated volume per formula unit variation with pressure for *Cmcm* LaSiH, *Pnma* LaSiH, and *Pnma* $LaSiH_2$, compared to the volumes from LeBail fitted models at 9 and 20 GPa (cf. Figs. S8 and S10).

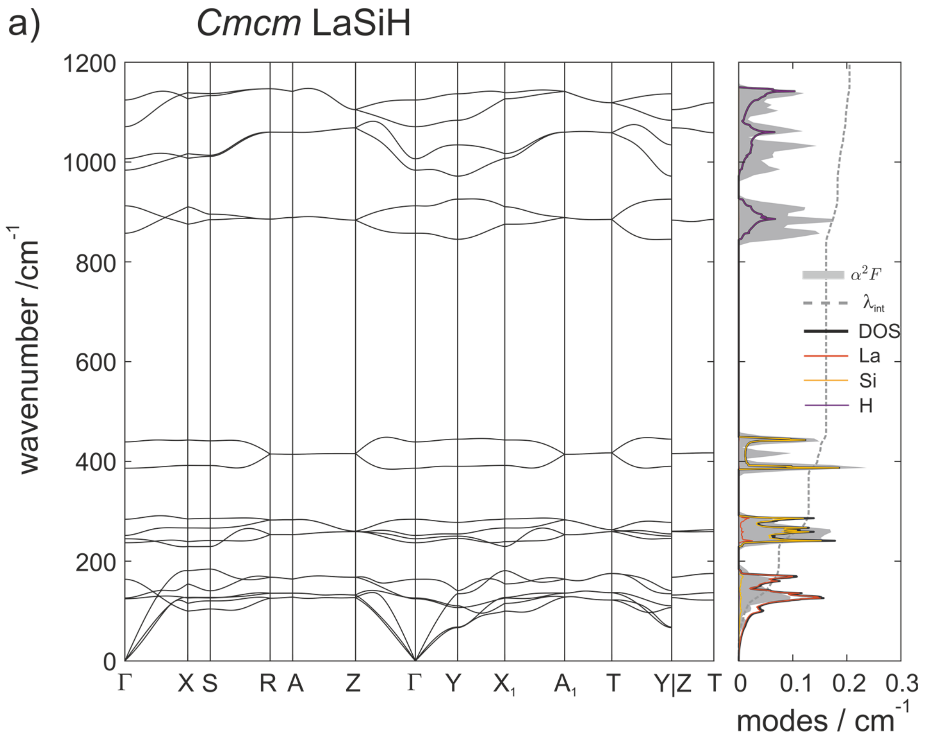

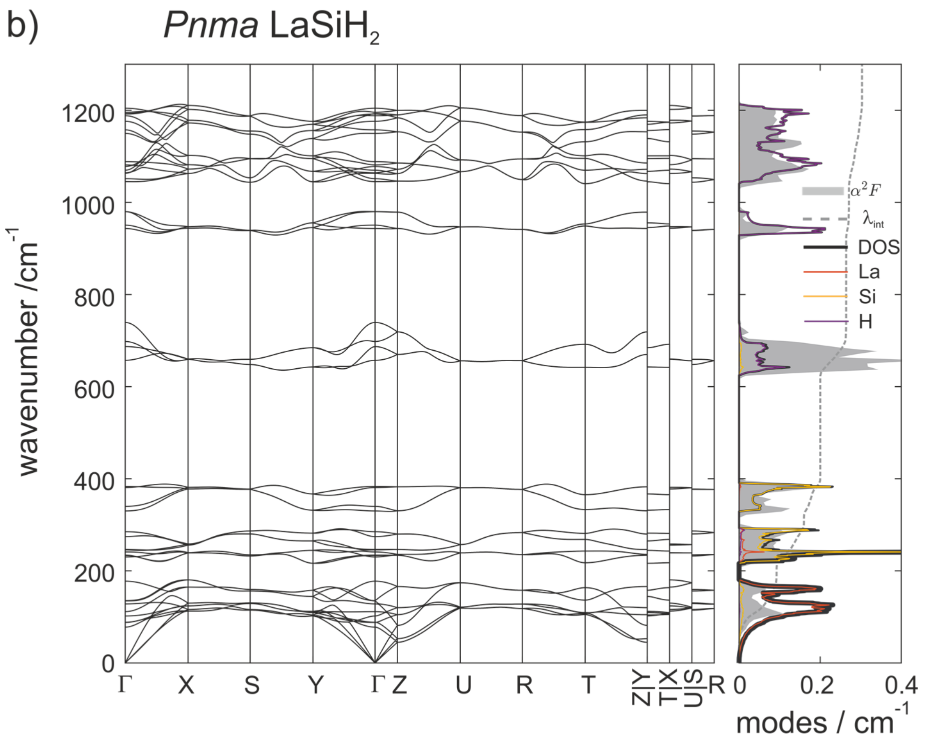

Figure S12. Phonon dispersions, phonon DOS (PDOS) decomposed into atomic contributions, and Eliashberg function $\alpha^2F(\omega)$ with electron-phonon coupling constant $\lambda(\omega)$ for *Cmcm*-LaSiH (a) and *Pnma*-$LaSiH_2$ (b) at 20 GPa.

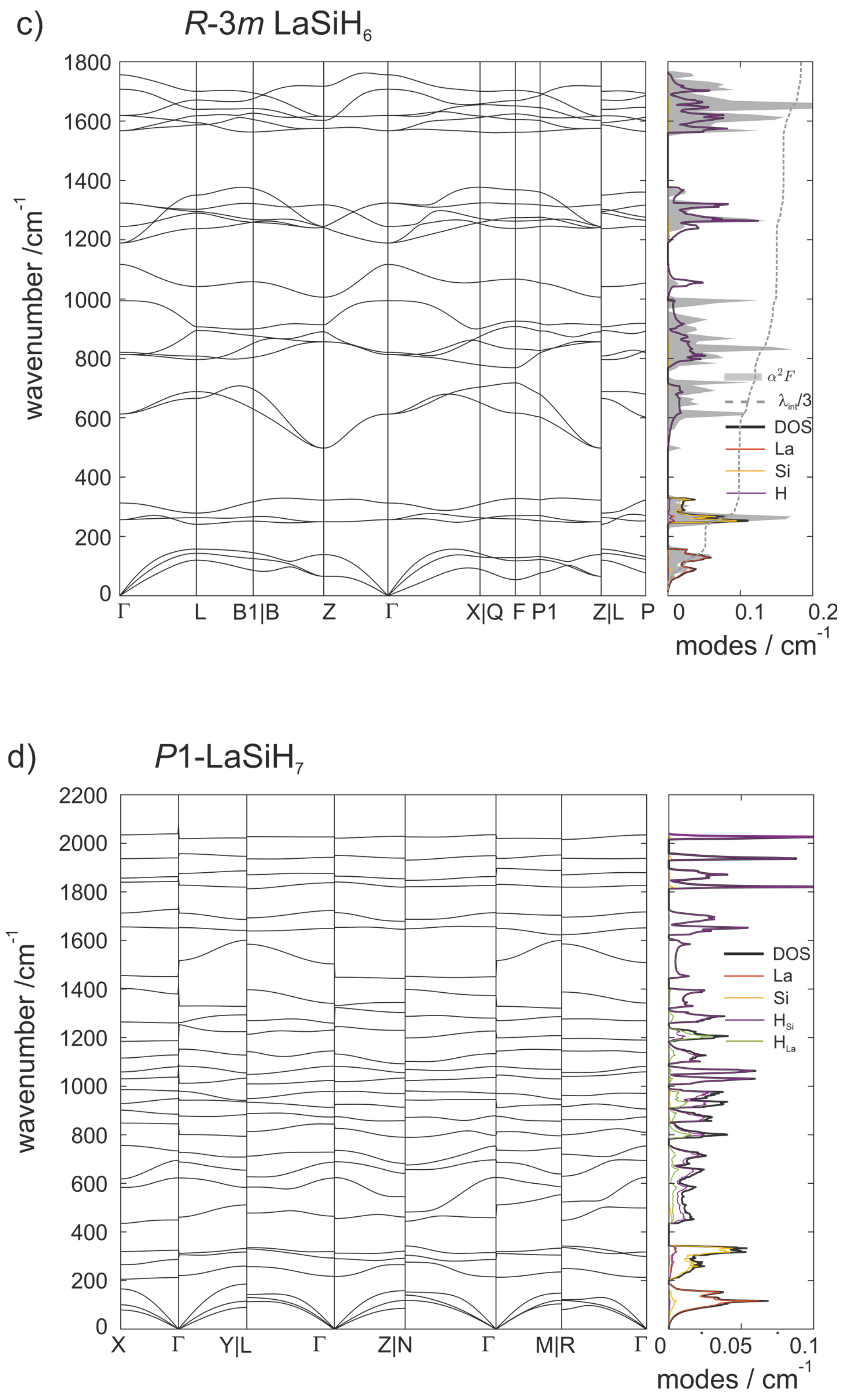

Figure S12, contd. Phonon dispersions, phonon DOS (PDOS) decomposed into atomic contributions, and Eliashberg function $\alpha^2F(\omega)$ with electron-phonon coupling constant $\lambda(\omega)$ for *R*-3*m*-$LaSiH_6$ (c) and *P*1-$LaSiH_7$ (d) at 20 GPa.

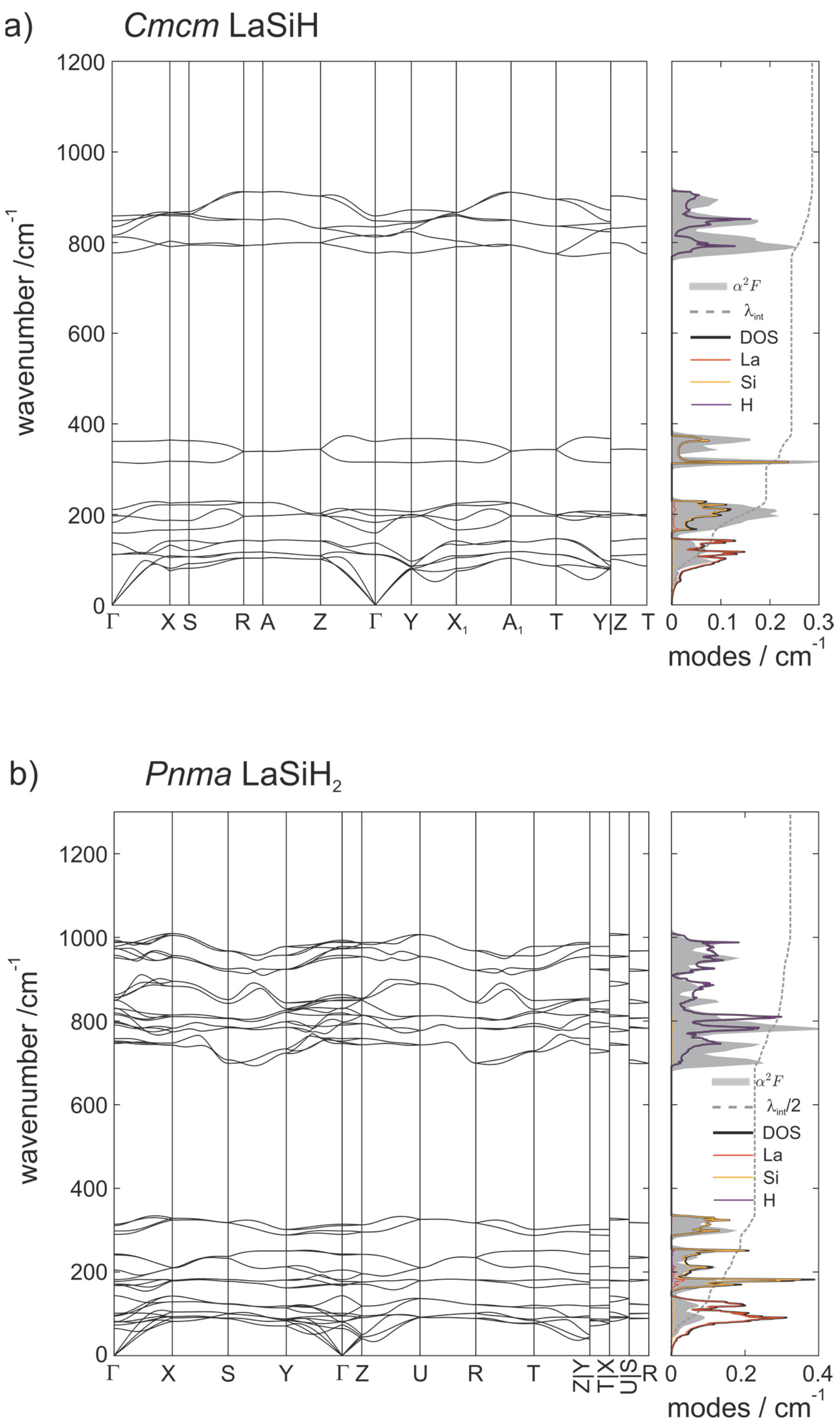

Figure S13. Phonon dispersions, phonon DOS (PDOS) decomposed into atomic contributions, and Eliashberg function $\alpha^2F(\omega)$ with electron-phonon coupling constant $\lambda(\omega)$ for *Cmcm* LaSiH (a) and *Pnma* $LaSiH_2$ (b), at 0 GPa.

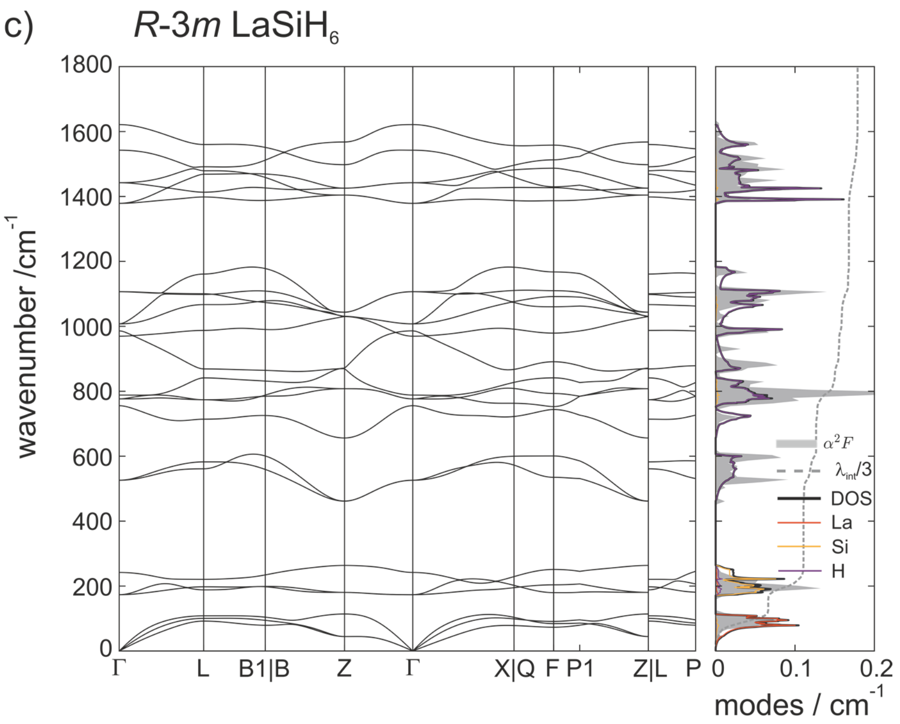

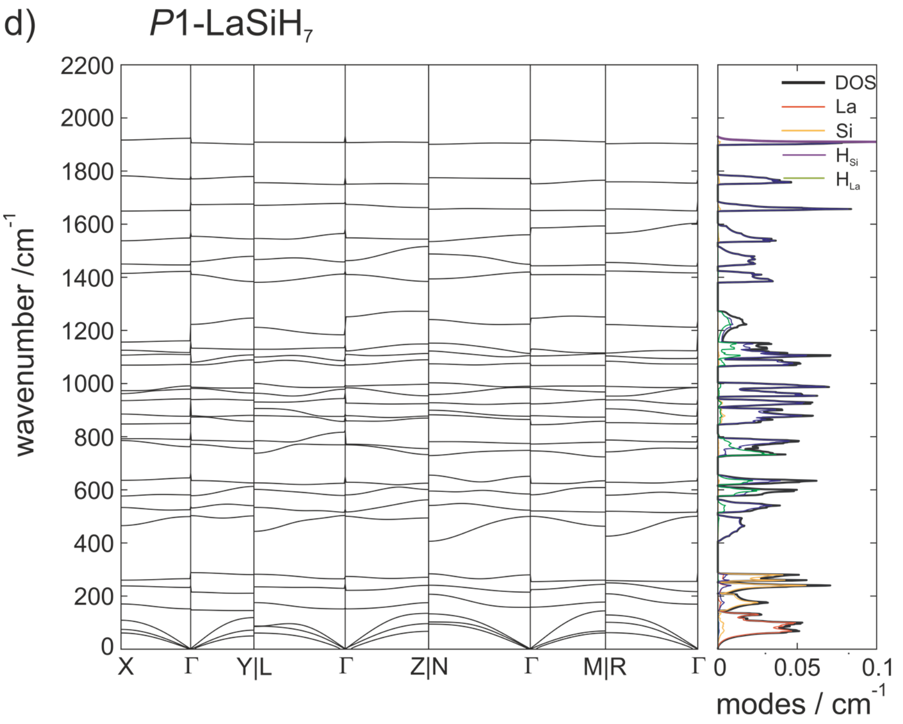

Figure S13. Phonon dispersions, phonon DOS (PDOS) decomposed into atomic contributions, and Eliashberg function $\alpha^2F(\omega)$ with electron-phonon coupling constant $\lambda(\omega)$ for *R*-3*m* $LaSiH_6$ (c) and *P*1 $LaSiH_7$ (d) at 0 GPa.

Table S2. Calculated structure data for *R*-3*m* and *R*-3 $LaAlH_6$ at 0 and 20 GPa.

	R-3*m* (0 GPa)	*R*-3*m* (20 GPa)	*R*-3 (0 GPa)	*R*-3 (20 GPa)
a / Å	6.5335	6.0395	6.5058	5.9496
c / Å	6.2988	5.9954	6.2910	5.9500
V / $Å^3$	232.8543	189.3898	230.5931	182.4015
Wyckoff positions	La1 3b (0, 0, 1/2)<br>Al1 3a (0, 0, 0)<br>H1 18h (0.5487, 0.4513, 0.1573)	La1 2d (1/3, 2/3, 0.13809)<br>La2 3b (0, 0, 1/2)<br>Al1 3a (0, 0, 0)<br>H1 18h (0.5459, 0.4541, 0.1527)	La1 3b (0, 0, 1/2)<br>Al1 3a (0, 0, 0)<br>H1 18f (0.5801, 0.4815, 0.1571)	La1 3b (0, 0, 1/2)<br>Al1 3a (0, 0, 0)<br>H1 18f (0.6215, 0.5251, 0.1535)

Table S3. Calculated structure data for *Cmcm* LaSiH at 0 and 20 GPa.

	0 GPa	20 GPa
a / Å	4.3167	4.1948
b / Å	12.2837	10.9375
c / Å	4.0508	3.8576
V / $Å^3$	214.7914	176.9856
Wyckoff positions	La1 4c (0, 0.6460, 1/4)<br>Si1 4c (0, -0.0546, 1/4)<br>H1 4c (0, 0.2434, 1/4)	La1 4c (0, 0.6488, 1/4)<br>Si1 4c (0, -0.0613, 1/4)<br>H1 4c (0, 0.2357, 1/4)

Table S4. Calculated structure data for *Pnma*-$LaSiH_2$ at 0 and 20 GPa.

	0 GPa	20 GPa
a / Å	12.2678	11.2279
b / Å	4.1486	3.990
c / Å	4.3233	4.0406
V / Å^3	220.0291	181.0177
Wyckoff positions	La1 4c (0.6463, 1/4, 0.5022)<br>Si1 4c (-0.0539, 1/4, 0.5061)<br>H1 4c (0.7500, 1/4, 0.0003)<br>H2 4c (-0.0465, 1/4, 0.1011)	La1 4c (0.6516, 1/4, 0.5018)<br>Si1 4c (-0.0595, 1/4, 0.5035)<br>H1 4c (0.7505, 1/4, 0.0006)<br>H2 4c (-0.0500, 1/4, 0.0680)

Table S5. Calculated structure data for *R*-3*m*-$LaSiH_6$ at 0 and 20 GPa.

	0 GPa	20 GPa
a / Å	4.3266	4.0701
c / Å	13.3309	12.1213
V / Å^3	216.1189	173.8991
Wyckoff positions	La1 3b (0, 0, 1/2)<br>Si1 (0, 0, 0)<br>H1 18h (0.8368, 0.1632, 0.2569)	La1 3b (0, 0, 0)<br>Si1 (0, 0, 1/2)<br>H1 18h (0.1529, 0.8471, 0.2450)

Table S6. Calculated structure data for *P*1 $LaSiH_7$ at 0 and 20 GPa.

	0 GPa	20 GPa
a / Å	4.2715	3.9828
b / Å	4.6059	4.1436
c / Å	4.7723	4.2230
α /°	94.7011	92.4093
β /°	116.5573	91.0703
γ /°	95.7330	116.1345
V / Å^3	82.7156	62.4584
Wyckoff positions	La1 1a (0.3665, 0.7595, -0.0917)<br>Si1 1a (0.6633, 0.3730, 0.4401)<br>H1 1a (0.8478, 0.6921, 0.4054)<br>H2 1a (0.0461, 0.2798, 0.5592)<br>H3 1a (0.5457, 0.2946, 0.0667)<br>H4 1a (-0.0761, -0.0392, -0.0272)<br>H5 1a (0.3172, 0.5372, 0.3292)<br>H6 1a (0.7809, 0.4910, 0.7994)<br>H7 1a (0.4775, 0.0627, 0.4379)	La1 1a (0.6673, 0.1395, 0.2071)<br>Si1 1a (0.3280, 0.5560, 0.6385)<br>H1 1a (0.1757, 0.6534, 0.3244)<br>H2 1a (-0.0762, 0.4400, 0.7439)<br>H3 1a (0.4816, -0.0303, 0.7077)<br>H4 1a (0.0707, -0.0455, 0.0035)<br>H5 1a (0.7075, 0.6730, 0.4574)<br>H6 1a (0.1882, 0.1615, 0.5018)<br>H7 1a (0.4883 0.5335, -0.0348)

Table S7. Bader charges and volumes for $LaSiH_n$ (n=1,2,6,7) at 0 and 20 GPa.

**0 GPa**	Bader z / |e|	Bader volume / Å^3
LaSiH		
La 4c	+1.483	147.4
Si 4c	-0.856	160.3
H 4c	-0.627	54.7
$LaSiH_2$		
La 4c	+1.528	140.9
Si 4c	-0.295	133.3
H1 4c (La_4)	-0.630	55.7
H2 4c (La_3Si)	-0.602	41.3
$LaSiH_6$		
La 3b	+1.613	143.9
Si 3a	+2.377	42.1
H 18h	-0.665	49.5
$LaSiH_7$		
La 1a	+1.800	137.9
Si 1a	+2.558	36.4
H1 1a (SiH_6)	-0.635	54.1
H2 1a	-0.547	61.2

**20 GPa**	Bader z / |e|	Bader volume / Å^3
LaSiH		
La 4c	+1.330	130.2
Si 4c	-0.767	129.1
H 4c	-0.563	39.3
$LaSiH_2$		
La 4c	+1.376	123.1
Si 4c	-0.245	109.6
H1 4c (La_4)	-0.573	39.4
H2 4c (La_3Si)	-0.558	33.3
$LaSiH_6$		
La 3b	+1.504	122.9
Si 3a	+2.463	35.0
H 18h	-0.661	38.9
$LaSiH_7$		
La 1a	+1.664	115.7
Si 1a	+2.617	31.3
H1 1a (SiH_6)	-0.632	38.2
H2 1a	-0.491	45.2

References

(1) Bulanova, M. V.; Zheltov, P. N.; Meleshevich, K. A.; Saltykov, P. A.; Effenberg, G.; Tedenac, J.-C. Lanthanum–silicon system. *J. Alloys Compd.* **2001**, *329* (1-2), 214–223. DOI: 10.1016/S0925-8388(01)01686-3.

(2) Raman, A.; Steinfink, H. The crystal structures of rare earth monosilicides. *Acta Cryst.* **1967**, *22* (5), 688–691. DOI: 10.1107/S0365110X67001367.

(3) Brauer, G.; Haag, H. Über Darstellung und Kristallstruktur der Disilicide von einigen Metallen der Seltenen Erden. *Z. Anorg. Allg. Chem.* **1952**, *267* (4-5), 198–212. DOI: 10.1002/zaac.19522670404.

(4) Coelho, A. A. TOPAS and TOPAS-Academic : an optimization program integrating computer algebra and crystallographic objects written in C++. *J. Appl. Cryst.* **2018**, *51* (1), 210–218. DOI: 10.1107/S1600576718000183.

(5) Coelho, A. A. *Topas Academic (version 5.0)*; Coelho Software, Brisbane, Australia, 2012.

(6) Zhou, S. H.; Napolitano, R. E. Phase equilibria and thermodynamic limits for partitionless crystallization in the Al–La binary system. *Acta Mater.* **2006**, *54* (3), 831–840. DOI: 10.1016/j.actamat.2005.10.013.

(7) Belyavina, N. M.; Markiv, V.; Zavodyanny, V. V. Crystal structure of the "La5Al4" compound. *J. Alloys Compd.* **2004**, *367* (1-2), 132–136. DOI: 10.1016/j.jallcom.2003.08.024.

(8) Leineweber, A.; Jacobs, H. Preparation of single crystals of LaAl and X-ray structure determination. *J. Alloys Compd.* **1998**, *278* (1-2), L10-L12. DOI: 10.1016/S0925-8388(98)00636-7.

(9) Lyaskovska, N.; Romaniv, O.; Semus'o, N.; Gladyshevskii, E. Crystal structures of the compounds RAl0.5−Si0.5+ (R = La, Ce, Pr, Nd, Sm, Gd), R3Al4Si6 (R = La, Pr), and RAlSi2 (R = Pr, Nd). *J. Alloys Compd.* **2004**, *367* (1-2), 180–184. DOI: 10.1016/j.jallcom.2003.08.033.

(10) Seto, S.; Nishio-Hamane, D.; Nagai, T.; Sata, N. Development of a software suite on X-ray diffraction experiments. *Rev. High Press. Sci. Technol.* **2010**, *20*, 269–276.

(11) Appa Rao, B.; Kistaiah, P.; Rajasekhar Reddy, N.; Satyanarayana Murthy, K. Thermal expansion of lanthanum dialuminide. *J. Mater. Sci. Lett.* **1982**, *1* (10), 432–434. DOI: 10.1007/BF00724863.

(12) Palenzona, A. The crystal structure and lattice constants of R.E.2In and some R.E.5In3 compounds. *J. Less-Common Met.* **1968**, *16* (4), 379–384. DOI: 10.1016/0022-5088(68)90135-5.

(13) Weidenthaler, C.; Pommerin, A.; Felderhoff, M.; Sun, W.; Wolverton, C.; Bogdanović, B.; Schüth, F. Complex rare-earth aluminum hydrides: mechanochemical preparation, crystal structure and potential for hydrogen storage. *J. Am. Chem. Soc.* **2009**, *131* (46), 16735–16743. DOI: 10.1021/ja9042565.
